# Network Pharmacology–Based Analysis and Experimental Exploration of Antidiabetic Mechanisms of Gegen Qinlian Decoction

**DOI:** 10.3389/fphar.2021.649606

**Published:** 2021-07-26

**Authors:** Yu Xu, Jihan Huang, Ning Wang, Hor-Yue Tan, Cheng Zhang, Sha Li, Guoyi Tang, Yibin Feng

**Affiliations:** ^1^ School of Chinese Medicine, Li Ka Shing Faculty of Medicine, The University of Hong Kong, Hong Kong, China; ^2^ Center for Drug Clinical Research, Shanghai University of Traditional Chinese Medicine, Shanghai, China

**Keywords:** type 2 diabetes mellitus, network analysis, herbal medicine, Gegen Qinlian decoction, multi-pathways

## Abstract

Type-2 diabetes mellitus (T2DM) and therapy options have been studied increasingly due to their rising incidence and prevalence. The trend of applying traditional Chinese medicine (TCM) to treat T2DM is increasing as a crucial medical care for metabolic dysfunctions. Gegen Qinlian decoction (GQL), a well-known classical TCM formula used in China, has been clinically applied to treat various types of chronic metabolic diseases. However, antidiabetic effects of GQL administration during T2DM have never been studied systematically. We assessed physiological and molecular targets associated with therapeutic effects of GQL by evaluating network topological characteristics. The GQL-related biological pathways are closely associated with antidiabetic effects, including the TNF and PI3K–AKT signaling pathways. Associated primary biological processes such as RNA polymerase II promoter transcription participate in the inflammatory response, oxidative stress reduction, and glucose metabolic process, thereby exerting multiple biological effects on the antidiabetic mechanism. Furthermore, our results showed that GQL can affect blood glycemic levels and ameliorate inflammatory symptoms, and liver and pancreas tissue injury in high-fat diet plus streptozotocin-induced diabetic mice. *In vivo* and *in vitro* experiments confirmed that antidiabetic effects of GQL were associated with a modulation of the TNF and PI3K–AKT–MTOR pathways.

## Introduction

Type-2 diabetes mellitus (T2DM) is a chronic metabolic disease which constitutes a severe threat to global public health (Chan et al., 2009). T2DM is a chronic hyperglycemia and inflammatory disease which may lead to macrovascular and microvascular degenerative complications, including cardiovascular diseases, diabetic nephropathy, and retinopathy (Usuelli and La Rocca, 2015). Metabolic dysfunction and inflammatory responses are partly responsible for islet function loss and irreversible host organ damage during pathological progression of T2DM and its associated complications. The World Health Organization estimated the number of deaths caused by diabetes mellitus and its related complications at approximately 1,600,000 in 2016. Severe diabetic complications and the lack of comprehensive therapy of T2DM and its associated afflictions require identification of novel effective treatment avenues (Kalra et al., 2018). As a crucial healthcare and alternative medicinal complement avenue, many traditional Chinese medicines (TCMs) have shown superior success regarding treatment of human metabolic disorders in China, Korea, and Japan. The remarkable efficacy and safety of TCMs with low toxicity have been acknowledged after several hundred years of practical clinical application in chronic metabolic disorder therapy (Pang et al., 2015).

As an alternative medicinal application, TCM has been demonstrated to exert excellent clinical effects on T2DM due to its rich herbal plant resources, many of which have been developed and are commonly used alone or in combination with adjuvant hypoglycemic agents for T2DM therapy (Covington, 2001). Gegen QinLian decoction (GQL) is a well-known classical TCM which has been used to treat chronic diarrhea and damp-heat syndrome, according to ancient records (Shang Han Lun) (Li et al., 2016). GQL is composed of four herbal drugs at a weight ratio of 5:3:3:2, for example, 15 g Puerariae Lobatae Radix (Gegen, *Pueraria lobata* (Wild.) Ohwi), 9 g Scutellariae Radix (Huangqin, *Scutellaria baicalensis* Georgi), 9 g Coptidis Rhizoma (Huanglian, *Coptis chinensis* Franch.), and 6 g Glycyrrhizae Radix et Rhizoma (zhi gan cao, *Glycyrrhiza uralensis* Fisch.). Modern clinical research has shown that GQL can normalize hyperglycemia and hyperlipidemia in T2DM patients (Ryuk et al., 2017), and its practical therapeutic application during diabetes mellitus has been assessed for more than ten years (Zeng et al., 2006). GQL was also used to treat T2DM-related complications with promising results (Han et al., 2017). However, the different constituents of GQL may exert various effects, which obscures the underlying molecular mechanisms; therefore, the respective antidiabetic chemical and pharmacological processes must be elucidated.

Systems pharmacology can help identify novel strategies and useful methods for discovering TCMs to treat complex diseases (Li and Zhang, 2013). In recent years, new network pharmacology combined with gene ontology (GO) enrichment analysis has become a useful tool to systemically determine interactions among TCM compounds, gene or protein targets, and pathways of diseases, which implies a holistic concept of TCM therapy (Li et al., 2014; Guo et al., 2019). Based on systemic bioinformatics, network pharmacology facilitates evaluation of feasibility and applicability of TCM for treating complex diseases through compound-target and target-signaling network analysis. Network pharmacology was successfully established in our laboratory for investigating complex herbal formulas used to treat human cancers (Wang et al., 2018b; Guo et al., 2019; Huang et al., 2020a). Meanwhile, previous ingredient–drug networks combined with GO biological analysis has showed that 4-hydroxymephenytoin from Puerariae Lobatae Radix can improve insulin metabolism in islet cells and adipocytes (Li et al., 2014). In the current study, we examined the pharmacological mechanisms of GQL and its effects on T2DM using network pharmacology analysis and experimental confirmation. Network pharmacology analysis was performed to identify the protective drug targets and the essential signaling pathways affected by GQL during T2DM. Furthermore, we examined GQL-related antidiabetic mechanisms using *in vivo* and *in vitro* experiments. Our results suggest the potential underlying mechanism of GQL and provide strong evidence for this therapeutic avenue of treating T2DM.

## Materials and Methods

### Ultrahigh-Performance Liquid Chromatography Analysis of Gegen Qinlian Decoction Constituents

Nong’s GQL formula (A190049310) was commercially purchased from PuraPharm (Hong Kong, China), and its constituents were identified using UHPLC analysis. GQL powder (1 g) was extracted using methanol (10 ml) in a 15-ml centrifuge tube, and the methanol solution was sonicated and centrifuged at 35,000 rpm. The supernatant solution was then filtered through a 0.45-μm membrane before UHPLC analysis. Chromatographic analysis was performed using a reverse-phase ACE Excel C18 column (100 mm × 2.1 mm) at a flow rate of 0.379 ml/min at 35 C. The elution media with 0.15% trifluoroacetic acid (B) and methanol (A) were used with a gradient protocol as follows: 77–70% B for 0–4.4 min, 70%–65% B for 4.4–4.576 min, 65%–42% B for 4.576–6.864 min, 42%–45% B for 6.864–7.040 min, 45%–45% B for 7.04–9.68 min, 45%–30% B for 9.68–12 min, and 30–30% B for 12–15 min.

### Interaction Network of Gegen Qinlian Decoction Constituents and T2DM Target Genes

We collected information on the GQL chemical constituents from previous studies (An et al., 2014; Wang et al., 2016; Qiao et al., 2016; Qiao et al., 2018). Forty-two active chemical GQL constituents were identified, including eight compounds from Puerariae Lobatae *Radix*, 14 compounds from Scutellariae Radix, seven compounds from Coptidis Rhizoma, and 13 compounds from Glycyrrhizae Radix et Rhizoma. Some chemical compounds of GQL show some properties of absorption, distribution, metabolism, and excretion (ADME) and the standard of drug-likeness (DL), and potentially druggable compounds were selected as active constituents for further target prediction of GQL. Information on oral bioavailability (OB) and DL index of the 42 compounds is shown in Supplementary Table S1. Bioinformatic data of protein targets of bioactive GQL constituents were analyzed based on the online TCMSP database (http://tcmspw.com/) (Supplementary Table S2). Next, T2DM-associated target genes were compiled using the TTD (http://db.idrblab.net/ttd/), KEGG (http://www.kegg.jp/), and CTD (http://ctdbase.org/) databases to identify corresponding T2DM-associated protein targets (Zhou et al., 2019). The complex network diagrams of active GQL constituents and identified anti-T2DM targets were plotted as an ingredient–target interaction network to be mapped using Cytoscape 3.6.1 software (Shannon et al., 2003). To illustrate GQL-related target protein interactions in T2DM, highly connected target proteins were screened based on protein–protein interaction information using STRING software (http://string-db.org). We used a high confidence threshold (>0.9) to ensure reliability, and protein–protein interaction data with high node degrees obtained from STRING were selected for establishing a protein–protein interaction network.

### Target Proteins Analysis

To identify potential target proteins affected by GQL, GO biological function and KEGG pathway enrichment searches were carried out using the annotation database of DAVID biological information (Huang et al., 2007) to determine the predicted target protein function affected by GQL and their important role in signaling transduction. The top 10 significantly enriched terms for the predicted proteins regarding biological process (BP), cellular components (CC), and molecular functions (MF) were produced using the Benjamini–Hochberg procedure (Haynes, 2013).

### Establishment and Treatment of a T2DM Mouse Model

All animal experiments were approved by the Committee on the Use of Live Animals in Teaching and Research of the University of Hong Kong. After adaptive feeding for one week, six-week-old C57BL/6J male mice were fed a high-fat diet (HFD, Research Diets, D12492) to induce obesity. After four weeks, HFD-fed mice were intraperitoneally injected with streptozotocin solution (STZ; Sigma-Aldrich, St. Louis, MO, United States ) at 50 mg/kg on two consecutive days (Srinivasan et al., 2005). Next, mice with high-fasting blood glucose levels (≥11.0 mmol/L) were randomly selected as T2DM models. The diabetic mice were assigned to a GQL treatment and a control group (*n* = 5, each). Following the best practice in pharmacological research (Heinrich et al., 2020), we used a common criterion of drug dosage, body surface area (BSA) formulas (Reagan-Shaw et al., 2008), to calculate the drug doses in mice. With respect to previous studies on GQL in clinical (Tong et al., 2011) and animal studies (Blanchard and Smoliga, 2015; Lv et al., 2019; Wu et al., 2019), we orally administered GQL at 1 g/kg (GQL-L group) and 2 g/kg (GQL-H group) to T2DM mice for six weeks. An oral glucose tolerance test (OGTT) was used to assess glucose tolerance. After an initial glucose gauge (2 g/kg), blood glucose levels were monitored using a glucometer at various time points. The glucose plot area under the curve (AUC) was used to evaluate glucose tolerance in T2DM mice after GQL treatment. The calculation of the glucose AUC was performed using Prism 8.3.1 software. Serum samples were used to measure insulin, HbA1c, ALT, and AST levels.

### Cell Culture

AML12 hepatocytes (obtained from the American Type Culture Collection, Manassas, VA, United States ) were cultured in DMEM/F12 medium (10% FBS plus 100 mg/ml streptomycin and 100 U/mL penicillin) and were incubated at 37 C in an incubator continuously supplying 5% CO_2_. To assess antidiabetic effects of GQL, we cultured AML12 cells in a palmitate plus high-glucose (33 mM) medium at 37 C for 24 h and treated the cells without or without GQL (100 μg/ml).

### Immunoblotting

Protein was extracted by adding RIPA solution, and supernatant containing proteins was collected after centrifugation. After protein quantification using a Bio-Rad Protein Assay Kit II (5000002), protein separation was performed by SDS-PAGE gel electrophoresis, and separated proteins were transferred to a membrane and blocked using 5% BSA solution to prevent nonspecific protein binding. After blocking, the immunoblot membrane was incubated with primary antibodies against the target proteins such as GAPDH, iNOS, P-65, SOCS 2, P-ERK, P-AKT, P-MTOR, and P-JNK. After washing three times, the membrane was incubated with secondary antibody (1:2,500). Protein expression was visualized using the ECL system and was analyzed using the Chemidoc chemiluminescent platform (Guo et al., 2019).

### RT-PCR Analysis

Total RNA was isolated by using an RNeasy Mini Kit (Qiagen, Hilden, Germany), and concentration was measured at a 260 /280-nm ratio. First-strand cDNA was transcribed from total RNA using a First Strand Synthesis Kit (Takara, Japan). PCR was conducted using SYBR Green reagent, specific primers (Table 1), and a Light Cycler 480 (Roche, Basel, Switzerland).

**TABLE 1:** PCR primer sequences and target genes.

Genes	Forward (5′-3′)	Reverse (5′-3′)
TNFα	CTA​CCT​TGT​TGC​CTC​CTC​TTT	GAG​CAG​AGG​TTC​AGT​GAT​GTA​G
IL-6	AGG​ATA​CCA​CTC​CCA​ACA​GAC​CT	CAA​GTG​CAT​CAT​CGT​TGT​TCA​TAC
IL1β	CTT​CAG​GCA​GGC​AGT​ATC​ACT​CAT	TCT​AAT​GGG​AAC​GTC​ACA​CAC​CAG
Caspase-8	CTC​CGA​AAA​ATG​AAG​GAC​AGA	CGT​GGG​ATA​GGA​TAC​AGC​AGA
Caspase-3	AAG​GAG​CAG​CTT​TGT​GTG​TGT	AAG​AGT​TTC​GGC​TTT​CCA​GTC
β-actin	ACG​GCC​AGG​TCA​TCA​CTA​TTG	TGG​AAA​AGA​GCC​TCA​GGG​C

### Hematoxylin and Eosin and Oil Red O Staining

Tissue samples of HFD + STZ mice were fixed in 4% paraformaldehyde buffer, and tissue sections (5 μm thickness) were stained using H&E and Oil Red O for general histology (Lucchesi et al., 2015).

### Data Analyses

Statistical analysis was conducted using an ANOVA or Student's two-tailed *t*-test with Prism Software 8.3.1. Statistical significance is reported at *p* < 0.05.

## Results

### Interaction Analysis of Gegen Qinlian Decoction and Type-2 Diabetes Mellitus Target Proteins

**FIGURE 1:**
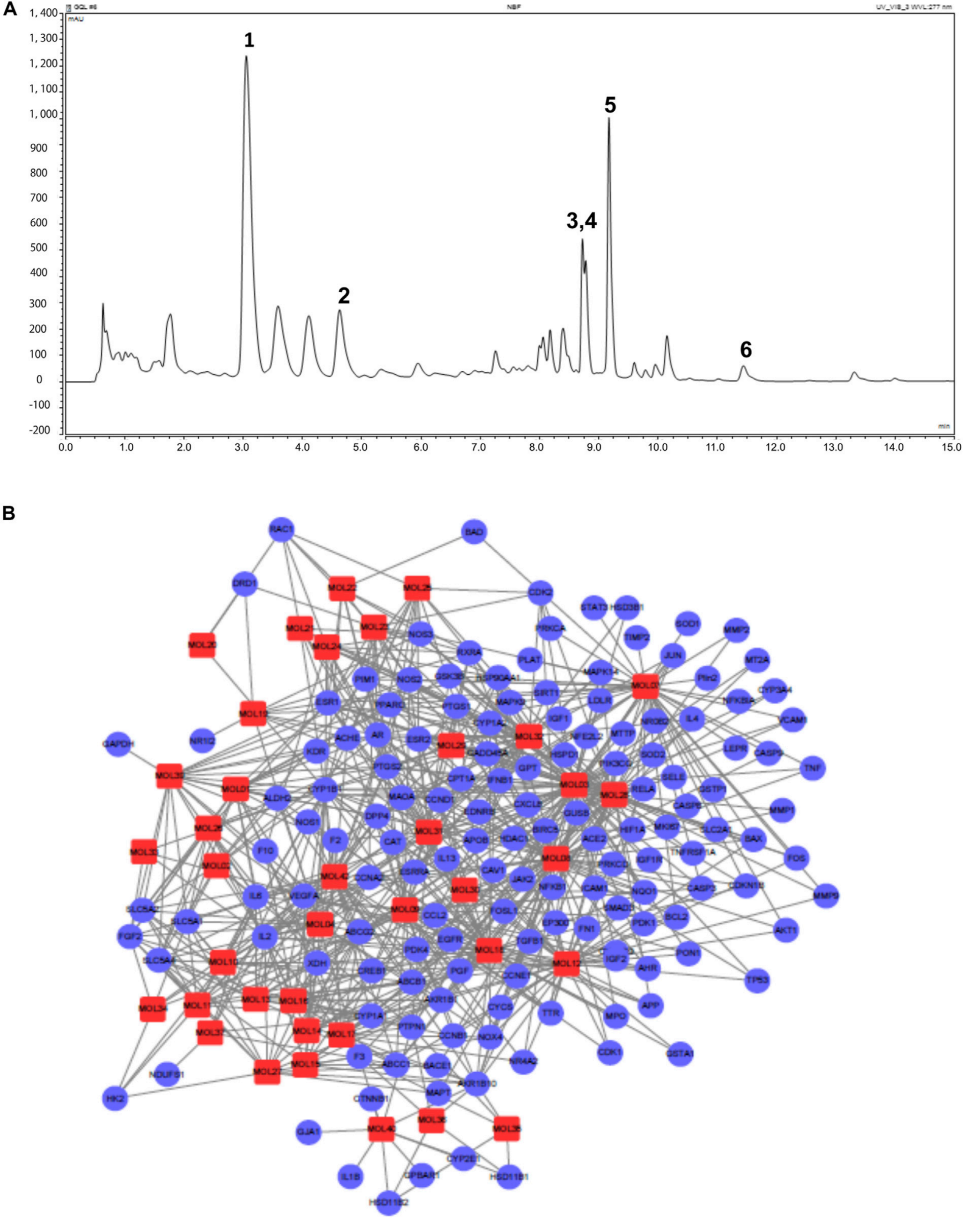
**(A)** UPLC-UV profiles of GQL. (1) Puerarin (MOL07), (2) daidzin (MOL01), (3) berberine (MOL24), (4) palmitane (MOL25), (5) baicalin (MOL17), and (6) baicalein (MOL12). **(B)** Compound–target network with 715 edges and 186 nodes. Red squares indicate antidiabetic active compounds (13) of GQL. Purple circles indicate 80 common target proteins of active GQL constituents and T2DM targets. Edges indicate interactions between targets and ingredients.

TCM was administered orally to examine its efficacy after the ADME process, and OB of the 42 active ingredients in GQL has been indicated to determine potentially druggable active compounds (Qiao et al., 2018). By screening the OB and DL index (Supplementary Table S1), we identified 12 candidate compounds from four herbs that contributed active compounds, including wogonin, oroxylin A, baicalein, baicalin, coptisine, epiberberine, berberine, palmatine, isoliquiritigenin, liquiritigenin, glycyrol, and formononetin (Supplementary Figure S1). Based on previous studies, GQL formula constituents were determined using UPLC analysis (Figure 1A), and puerarin (MOL07), daidzin (MOL01), berberine (MOL24), palmitane (MOL25), baicalin (MOL17), and baicalein (MOL12) were identified as the major active constituents of the GQL formula. GQL contained puerarin at 1.038%, which meets the quality standard requirement of GQL formula according to the China Pharmacopeia 2015. We explored the therapeutic targets of the 42 active GQL constituents, and SMILES structural similarity of the selected active GQL constituents (Supplementary Table S3) was used to investigate the drug–target interaction prediction through the similarity ensemble approach, in which 38 different ingredients of GQL with practical pharmacological activities were closely associated with the 468 target proteins shown in Supplementary Table S4. Accordingly, the component–target–disease network showed that 38 active ingredients interacting with 148 T2DM-related target genes were generated using a therapeutic target database (Wang et al., 2020), and the network visualization was analyzed using Cytoscape 3.6.1 (Figure 1B). The compound–target network comprising 715 edges and 186 nodes showed that six high-degree compounds were associated with multiple target proteins, that is, MOL18 (chrysin, 38), MOL12 (baicalein, 44), MOL03 (daidzein, 58), MOL07 (puerarin, 41), MOL08 (wogonin, 53), and MOL28 (isoliquiritigenin, 46). Critical target proteins or ingredients with a high degree of connection in the interaction network may be responsible for essential antidiabetic effects of GQL.

### Gegen Qinlian Decoction Type-2 Diabetes Mellitus Target Protein Identification

**FIGURE 2:**
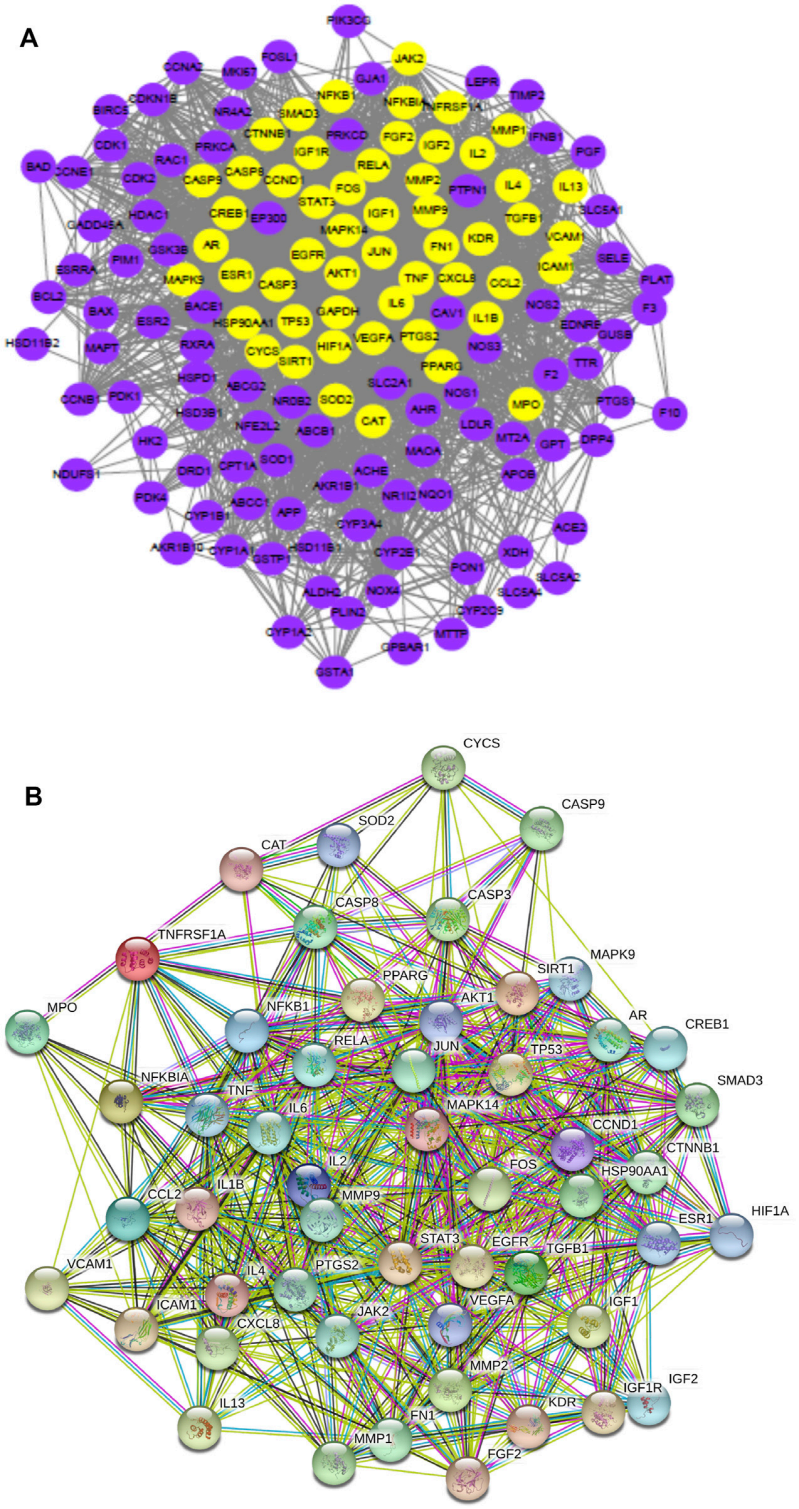
**(A)** Highly connected subnetworks (53 nodes, 1,120 edges) produced using Cytoscape 3.6.0, **(B)** a Venn diagram of the four herb compound-related gene numbers, and **(C)** protein–protein interaction network (52 nodes, 296 edges) produced using STRING.

**FIGURE 3:**
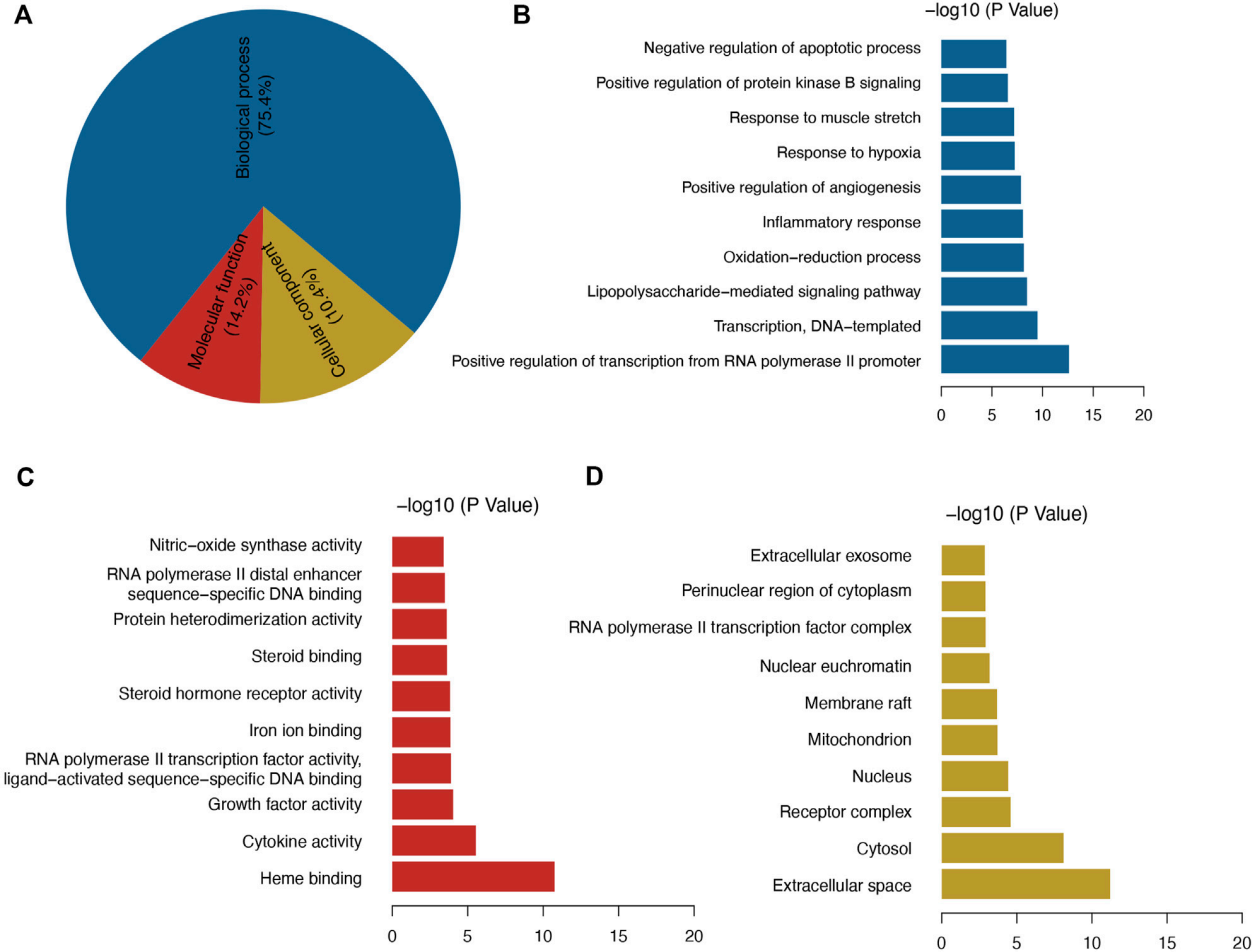
**(A)** GO enrichment analysis, and **(B–D)** top 10 significant GO enrichment terms in categories BP, MF, and CC.

Highly connected subnetworks with 53 gene nodes were identified using the MCODE module analysis (Zhang et al., 2019) (Figure 2A). The Venn diagram results (Figure 2B) suggested 11 overlapping genes which were identified by matching the four herbal compounds-related genes with each other, including AR, ESR1, PTGS2, PIM1, CDK2, ESR2, HSP90AA1, NOS2, NOS3, PTGS1, and RXRA. This interaction network contained 296 edges and 52 nodes (Figure 3C), of which edges represent interactions between the proteins and nodes represent target proteins. MAPK14, JUN, STAT3, IL-2, JAK2, TP53, CCND1, AKT1, FOS, RELA,MMP9, SIRT1, PPARG, IL-6, EGFR, TGFB1, VEGFA, JAK2, PTGS2, IL1B, TNF, and NFKB1 were centrally located in the interaction network with high node degrees, suggesting that these high-degree proteins may be the key antidiabetic targets of GQL during T2DM treatment.

### Identification of Potential Signaling Pathways

**FIGURE 4:**
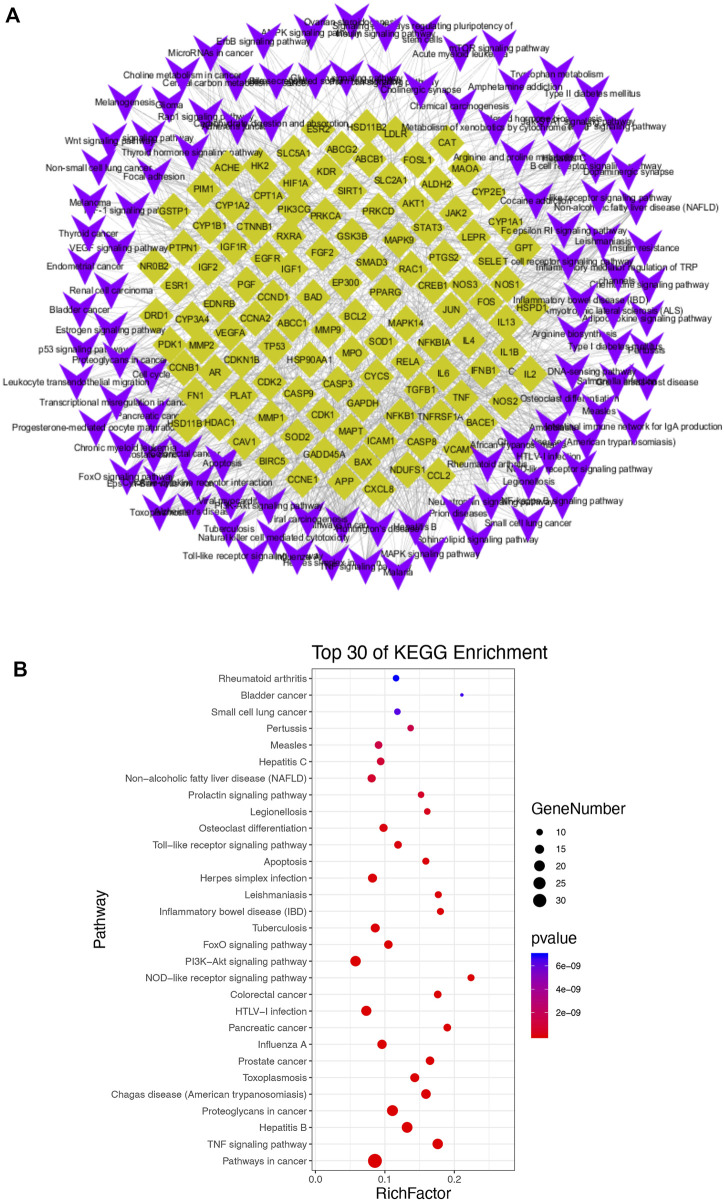
**(A)** Target–pathway interaction network and **(B)** KEGG enrichment analysis.

Among the GO enriched categories, the BP ontology (101 records), CC ontology (14 records), and MF ontology (19 records) consisted of 75.4, 14.2, and 10.4%, respectively (Figure 3A). Target proteins of the BP category were mostly associated with RNA polymerase II promoter transcription and were relevant to the inflammatory response, oxidative stress reduction, and glucose metabolic process (Figure 3B). Target proteins in the MF category were predominantly associated with heme binding and cytokine activity (Figure 3C), and CC target proteins were categorized as belonging to extracellular space or cytosol (Figure 3D). The results showed that GQL may thus bind kinase in plasma or in the cell membrane during inflammatory response, oxidative stress, and glucose metabolism. To further elucidate the association of target proteins with signaling pathways, a target–pathway interaction network was produced based on GQL-related target proteins (Figure 4A). Furthermore, the top 30 KEGG pathways were screened out (Benjamini–Hochberg corrected *p* < 0.05) to generate a target–pathway signaling network involving 33 target proteins (Figure 4B). KEGG analysis indicated that these target proteins mostly participated in the regulation of TNF, NOD-like receptor, PI3K–AKT, FoxO, TLR, and apoptosis. GO and KEGG enrichment analyses suggested that bioactive ingredients of GQL affecting the TNF inflammatory signaling and PI3K/AKT pathways were responsible for the main therapeutic effects during T2DM.

### Antidiabetic Effects of Gegen Qinlian Decoction in Diabetic Mice

**FIGURE 5:**
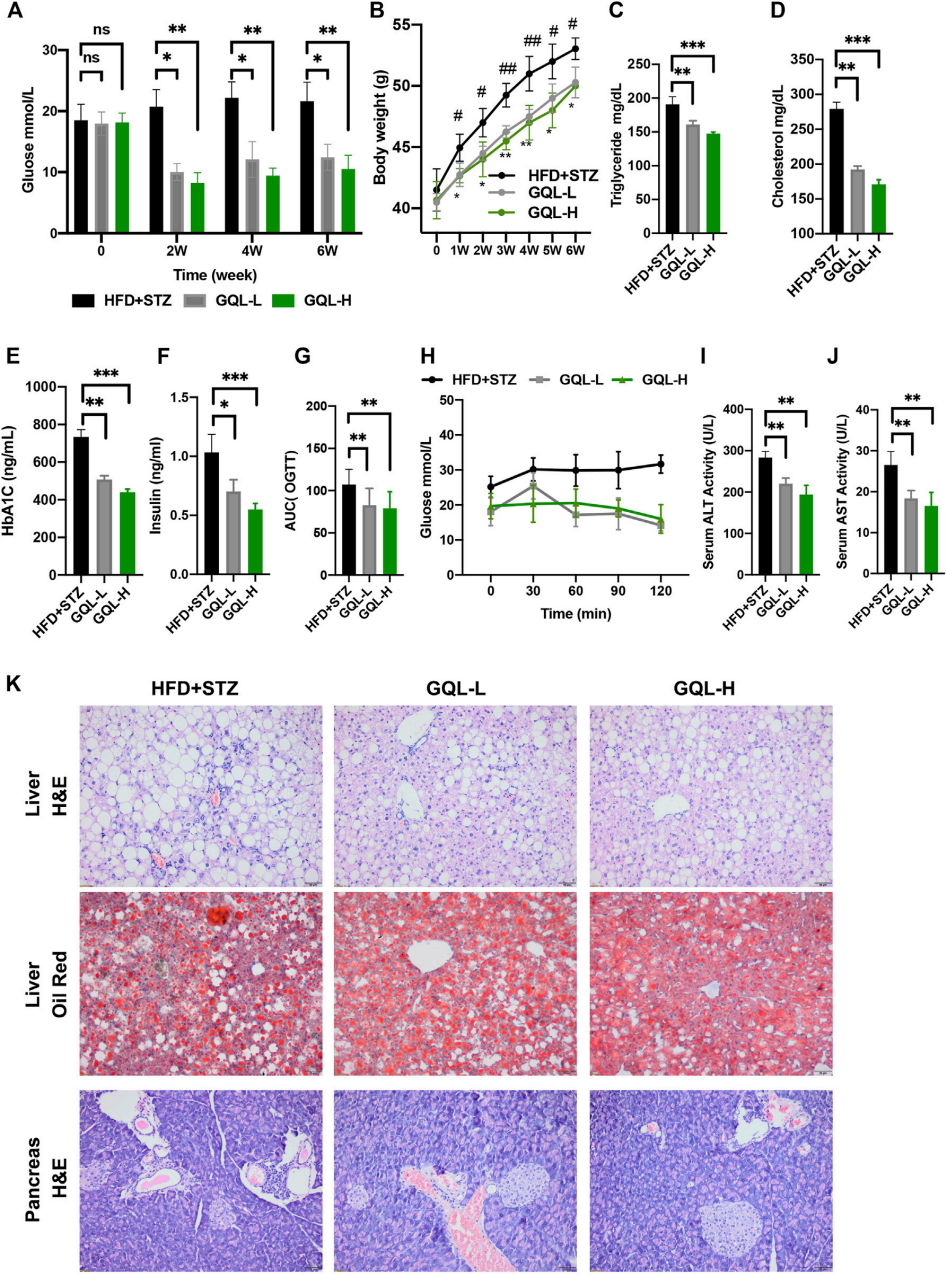
**(A)** Fasting blood glucose changes in HFD + STZ-treated C57BL/6 mice after GQL treatment; **(B)** body weight elevation; **(C,D)** triglyceride and cholesterol levels; **(E,F)** HbA1c and insulin levels; **(G)** glucose AUC during OGTT; **(H)** glucose levels during OGTT measured 0, 30, 60, 90, and 120 min after oral administration of glucose solution (2 g/kg); **(I,J)** serum ALT and AST levels.

**FIGURE 6:**
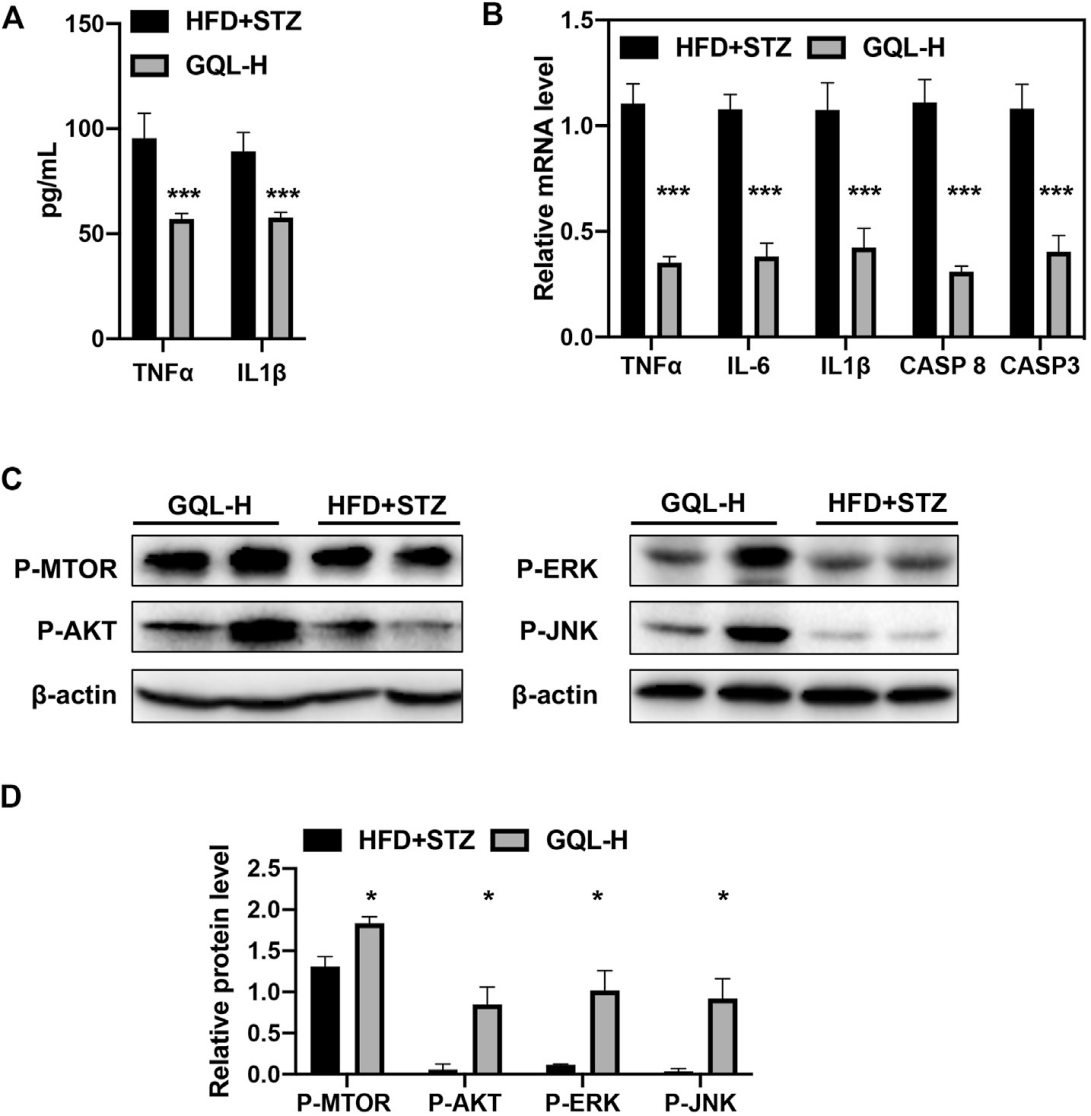
**(A)** Serum TNFα and IL1β; **(B)** levels of TNFα, IKKα, IL-6, IL1β, CASP 8, CASP3, and AP-1 mRNA in diabetic mice determined using RT-PCR; **(C,D)** relative levels of P-AKT, P-MTOR, -JNK, and P-ERK proteins detected by immunoblotting.

Curative effects of the GQL formula on HFD + STZ-induced diabetic mice were evaluated, and GQL treatment showed promising hyperglycemic effects, as evidenced by the reduction in fasting blood glucose levels (Figure 5A). Compared with the controls, the GQL-L and GQL-H treatment mice showed significantly reduced glucose levels during the six-week treatment. Moreover, improvements regarding body weight (Figure 5B), triglycerides (Figure 5C), cholesterol (Figure 5D), HbA1C (Figure 5E), and insulin levels (Figure 5F) were also observed in the GQL treatments. The OGTT assay showed lower AUC values in GQL-L GQL-H mice than in the controls (Figures 5G,H). GQL treatment significantly reduced the levels of ALT and AST in the serum of HFD + STZ mice (Figures 5I,J). Severe steatosis and cytoplasmic vacuoles were observed in hepatocytes of HFD + STZ mice, in addition to inflammatory infiltration. Oral administration of GQL prevented fat deposition in liver tissues, as shown by Oil Red O staining (Figure 5K). Compared with the controls, liver structures were significantly altered in GQL-L and GQL-H mice, as indicated by smaller amounts of fatty vacuoles and less interlobular mononuclear inflammation (Figure 5K). Under hyperglycemic conditions, the pancreatic tissue of HFD + STZ model mice showed severe necrotic changes and a reduction in the size of islets, especially around large vessels (Pandey et al., 2019). GQL supplementation prevented histomorphological changes in pancreatic tissues of HFD + STZ mice. These results suggested that GQL alleviates liver and islet cell damage in pancreatic tissues caused by diabetes mellitus. By examining antidiabetic effects of GQL in HFD + STZ mice, we found that GQL reduced the serum levels of TNFα and IL-1β (Figure 6A) and the levels of TNF-α, IKKα, IL-6, IL-1β, CASP 8, and CASP three mRNA (Figure 6B), which confirmed that antidiabetic effects of GQL were associated with TNF-α signaling. Additionally, GQL increased the levels of phosphorylated AKT, MOTR, ERK, and JNK in liver tissue (Figures 6C,D), confirming activation of AKT/mTOR signaling in GQL-treated diabetic mice.

### *In vitro* Confirmation of PI3K/AKT and TNF-α Signaling Pathway Activation by Gegen Qinlian Decoction

**FIGURE 7:**
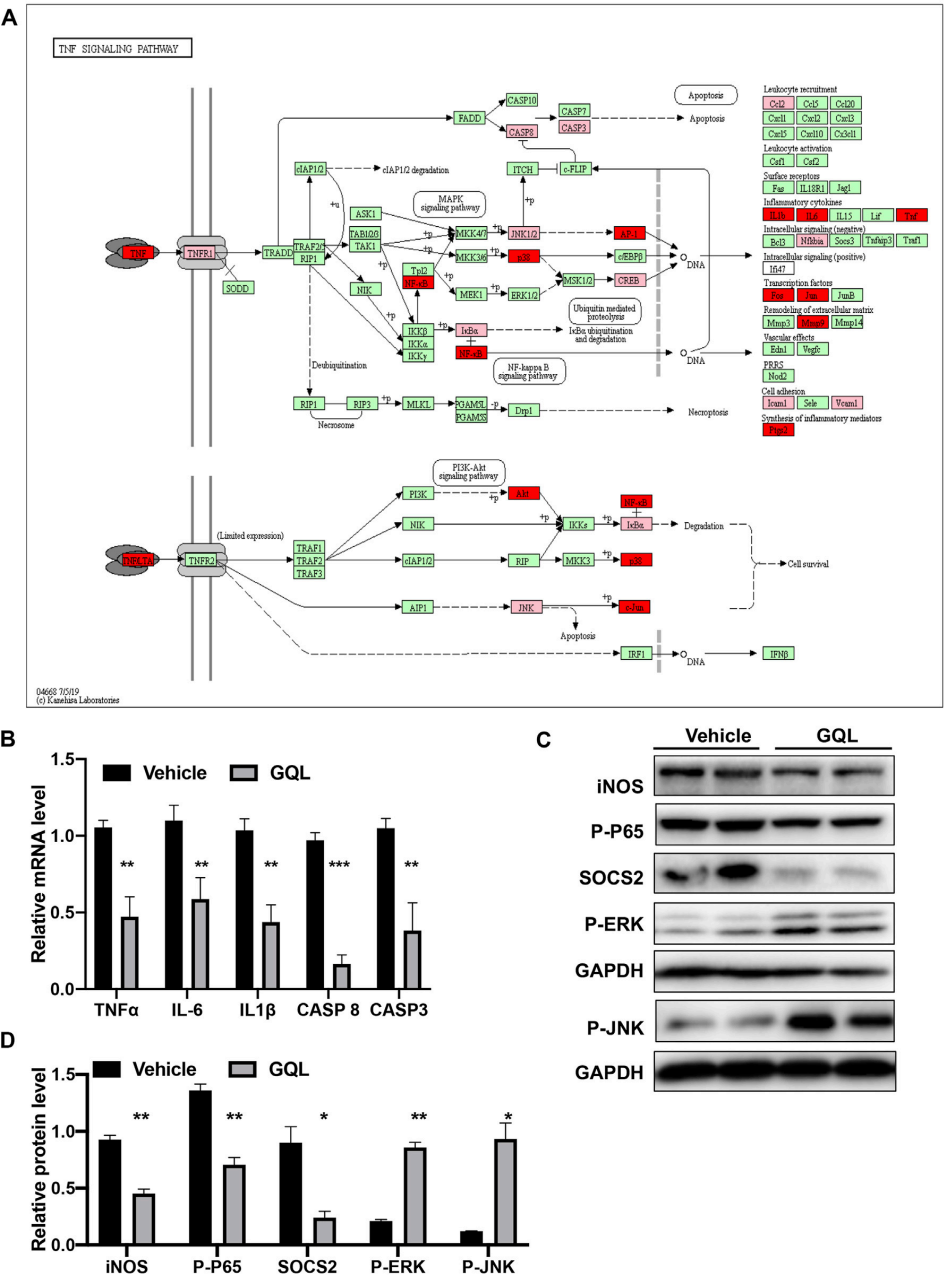
**(A)** KEGG pathway map of the TNFα signaling pathway; **(B)** levels of TNFα, IKKα, IL-6, IL1β, CASP 8, CASP3, and AP-1 mRNA determined using RT-PCR; **(C,D)** relative expression of iNOS, P-P65, SOCS2, P-JNK, and P-ERK as detected by immunoblotting.

**FIGURE 8:**
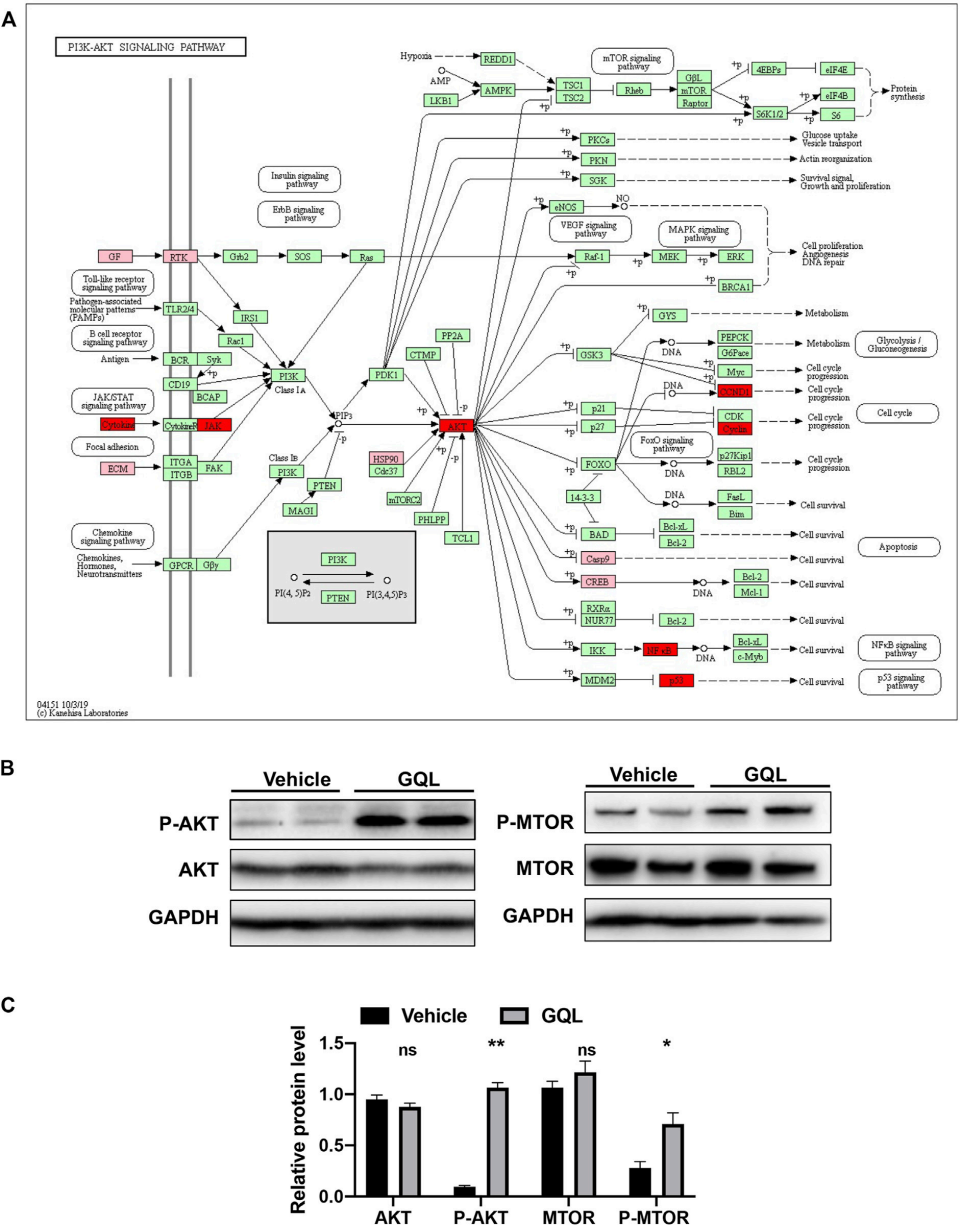
**(A)** KEGG pathway map of the AKT signaling pathway, and **(B)** relative levels of P-AKT, AKT, P-MTOR, and MTOR proteins as evaluated by immunoblotting.

PI3K/AKT and TNF-α–related target proteins and signaling pathways were assessed in AML12 hepatocytes exposed to palmitate and high glucose levels. The TNF signaling pathway was examined by measuring TNFα, IKKα, IL-6, IL-1β, CASP 8, and CASP 3 mRNA (Figure 7A), showing that GQL treatment significantly reduced TNF-related inflammatory proteins, including TNFα, IKKα, IL-6, and IL-1β (Figure 7B). Furthermore, GQL-induced inflammation reduction was associated with reduced levels of phosphorylated NF-κB protein and increased phosphorylated JNK and ERK1/2 production (Smith et al., 2007), as well as SOCS3 protein expression, which contributed to apoptosis (Figures 7C,D). To confirm activation of the PI3K/AKT signaling pathway (Figure 8A), we measured AKT and MTOR proteins in AML12 cells using Western blotting. GQL treatment increased AKT and mTOR phosphorylation (Figures 8B–D), suggesting that GQL may activate AKT/mTOR signaling.

## Discussion

T2DM is a heterogeneous disease with high morbidity and complex associated afflictions. Thus, development of diabetes is associated with multiple target proteins or pathways. TCMs composed of multiple indigents may exert various pharmacological effects *via* multiple targets and signaling pathways, which may aid in T2DM treatment. However, the complexity of TCMs may complicate in-depth research to elucidate the underlying mechanisms. Due to extensive clinical application of GQL to treat T2DM in China (Ryuk et al., 2017), network pharmacology is important to verify the pharmacological mechanism by which GQL attenuates T2DM.

Our study explored the mechanisms of a multicomponent, multigene-targeting GQL formula by establishing a T2DM association network. This analysis was based on therapeutic effects of GQL formula relevant to 148 antidiabetic target genes, and GQL regulated binding kinase in plasma or cell membranes and modulated inflammatory responses, oxidative reduction, and glucose metabolic process by 38 significant proteins and signaling pathways, that is, the PI3–AKT (Huang et al., 2018), insulin (Brännmark et al., 2013), and TNF signaling pathways (Alipourfard et al., 2019). Based on KEGG pathway analysis, PI3-AKT and TNF signaling pathways were among the top 30 significant pathways, and GO enrichment highlighted the main biological processes of RNA polymerase II promoter transcription for modulating insulin metabolism, glucose homeostasis, and inflammation, thereby exerting multicomponent, multi-target, multichannel, and antidiabetic effects. Chronic low-grade inflammation is a common characteristic of T2DM, with primary alterations occurring in the liver and pancreatic islets. The NF-κB and JNK signaling pathways contribute to chronic inflammation during T2DM. GQL can be used to treat inflammation and oxidative stress so as to reduce the severity of colitis by inhibiting TLR4/NF-κB activation (Li et al., 2016). Our experiments indicated that GQL suppressed activation of NF-κB and of two major mitogen-activated protein kinases (ERK1/2 and JNK). Inhibition of NF-κB and ERK1/2 ameliorated TNF-α–induced inflammation induced by high glucose exposure (Smith et al., 2007). Our results showed that GQL promoted phosphorylation of AKT and mTOR *in vitro* and *in vivo* to inhibit T2DM development.

In the current study, GQL constituents with high OB and DL indices were selected as bioactive compounds with high pharmacokinetics because they may be absorbed and distributed in the patient’s body. Our compound–target network analysis showed 12 potential candidate compounds with high node degrees, that is, wogonin, oroxylin A, baicalein, baicalin, coptisine, epiberberine, berberine, palmatine, isoliquiritigenin, liquiritigenin, glycyrol, and formononetin, all of which may be associated with the marked antidiabetic effect of GQL on T2DM. These bioactive compounds exert antihyperlipidemic, anti-inflammatory, and anti-oxidative effects during diabetes and diabetic complications. Bioactive isoflavones from Puerariae Lobatae Radix, puerarin, and daidzin showed antidiabetic effects in animal studies, such as puerarin improving insulin resistance and islet damage by inhibiting inflammation and oxidative stress during diabetes and diabetic complications (Chen et al., 2018). Daidzin can modulate glucose and lipid metabolism and reduces the inflammatory response through the TNFα/JNK signaling pathway in macrophages during T2DM (Das et al., 2018). Wogonin, baicalin, and baicalein are active ingredients of Scutellariae Radix, which have been considered potential anti-oxidative and anti-inflammatory agents for treating obesity, insulin resistance, and inflammatory disorders (Fang et al., 2020). Wogonin can increase glucose cellular absorption to reduce hyperglycemia through the AKT and GLUT4 pathways (Khan and Kamal, 2019), and it exerts anti-inflammatory effects through NF-κB signaling and anti-fibrosis effects against diabetic nephropathy through the TGF-β1/Smad3 signaling pathway (Zheng et al., 2020); moreover, it can alleviate diabetic cardiomyopathy through anti-inflammatory and anti-oxidative activities (Khan et al., 2016a). Alkaloids, especially berberine, palmatine, and coptisine, are responsible for therapeutic effects of *Rhizoma coptidis*, which can have beneficial effects on diabetes and diabetic complications by modulating AKT/AMPK–NF-κB/MAPK/PI3K and oxidative stress signaling pathways (Wang et al., 2018a). Specifically, berberine acts as an antihyperglycemic agent during T2DM treatment through increased phosphorylation of AKT, thereby improving insulin resistance through AMPK activation (Chang et al., 2015). Coptisine ameliorates oxidative injury in diabetic nephropathy by regulating the Nrf2 signaling pathway, and liquiritigenin inhibits diabetes-induced mesangial matrix accumulation in diabetic nephropathy by decreasing the NF-κB and NLRP3 inflammasome (Zhu et al., 2018). Isoliquiritigenin attenuates inflammation and oxidative stress in diabetic renal injuries through an SIRT1-dependent mechanism (Huang et al., 2020b). These previous studies on bioactive compounds and the results of the present study support the use of pharmacological network prediction. In the exploration of the potential underlying mechanism, network pharmacology has shaped its own analytical rules and evaluation of rationality (Li, 2021). Our results suggested successful network pharmacology for screening the mechanism of action of TCMs with respect to a specific disease. Anti-inflammation and PI3K-AKT/MTOR activation (Khan et al., 2016b) and multiple active ingredients of GQL that can synergize with numerous target proteins result in diverse beneficial mechanisms in the treatment T2DM. Our results showed that the antihyperglycemic effects of GQL were associated with alleviation of liver and pancreas injury, which is common during T2DM (Loria et al., 2013). Moreover, our results indicated that GQL in T2DM treatment may affect the TNF/NF-κB and PI3K/AKT/MTOR pathways to reduce inflammation and improve hyperglycemia. Furthermore, detailed pharmacological mechanisms by which GQL ameliorates T2DM will be investigated in our future study.

In conclusion, a combination of pharmacology network analysis and experimental approaches may be a useful research tool to elucidate antidiabetic mechanisms of TCMs in detail. The development of T2DM is a complex pathological process involving multiple signaling pathways and multiple targets. Our results suggest that GQL can modulate multiple target proteins in multiple signaling pathways and can be used for T2DM therapy. The antidiabetic effects of GQL *in vitro* and *in vivo* should be further examined in clinical trials with T2DM patients. However, more evidence is needed to further validate antidiabetic bioactivities of the active compounds and to evaluate their respective contributions.

## Data Availability

The original contributions presented in the study are included in the article/Supplementary Material; further inquiries can be directed to the corresponding author.

## References

[B1] Alipourfard I. Datukishvili N. Mikeladze D. (2019). TNF-α Downregulation Modifies Insulin Receptor Substrate 1 (IRS-1) in Metabolic Signaling of Diabetic Insulin-Resistant Hepatocytes. Mediators Inflamm. 2019, 3560819. 10.1155/2019/3560819 30863203 PMC6378771

[B2] An R. You L. Zhang Y. Wang X. Ma Y. (2014). A Rapid UPLC Method for Simultaneous Determination of Eleven Components in ‘Ge-Gen-Qin-Lian' Decoction. Pharmacogn. Mag. 10, 464. 10.4103/0973-1296.141821 25422547 PMC4239724

[B3] Bak E. J. Kim J. Choi Y. H. Kim J. H. Lee D. E. Woo G. H. (2014). Wogonin Ameliorates Hyperglycemia and Dyslipidemia via PPARα Activation in Db/db Mice. Mol. Cel Endocrinol 33, 156. 10.1016/j.clnu.2013.03.013 23623334

[B4] Blanchard O. L. Smoliga J. M. (2015). Translating Dosages from Animal Models to Human Clinical Trials-Revisiting Body Surface Area Scaling. FASEB j. 29, 1629–1634. 10.1096/fj.14-269043 25657112

[B5] Brännmark C. Nyman E. Fagerholm S. Bergenholm L. Ekstrand E.-M. Cedersund G. (2013). Insulin Signaling in Type 2 Diabetes. J. Biol. Chem. 288, 9867–9880. 10.1074/jbc.m112.432062 23400783 PMC3617287

[B6] Chan J. C. N. Malik V. Jia W. Kadowaki T. Yajnik C. S. Yoon K.-H. (2009). Diabetes in Asia. JAMA 301, 2129–2140. 10.1001/jama.2009.726 19470990

[B7] Chang W. Chen L. Hatch G. M. (2015). Berberine as a Therapy for Type 2 Diabetes and its Complications: From Mechanism of Action to Clinical Studies. Biochem. Cel Biol 93, 479. 10.1139/bcb-2014-0107 25607236

[B8] Chen X. Yu J. Shi J. (2018). Management of Diabetes Mellitus with Puerarin, a Natural Isoflavone FromPueraria Lobata. Am. J. Chin. Med. 46, 1771–1789. 10.1142/s0192415x18500891 30525896

[B9] Covington M. B. (2001). Traditional Chinese Medicine in the Treatment of Diabetes. Diabetes Spectr. 14, 154–159. 10.2337/diaspect.14.3.154

[B10] Das D. Sarkar S. Bordoloi J. Wann S. B. Kalita J. a.-O. Manna P. a.-O. (2018). Daidzein, its Effects on Impaired Glucose and Lipid Metabolism and Vascular Inflammation Associated with Type 2 Diabetes. Biofactors 44, 407. 10.1002/biof.1439 30191623

[B11] Fang P. Yu M. Shi M. Bo P. Gu X. Zhang Z. (2020). Baicalin and its Aglycone: a Novel Approach for Treatment of Metabolic Disorders. Pharmacol. Rep. 72, 13–23. 10.1007/s43440-019-00024-x 32016847

[B12] Guo W. Huang J. Wang N. Tan H.-Y. Cheung F. Chen F. (2019). Integrating Network Pharmacology and Pharmacological Evaluation for Deciphering the Action Mechanism of Herbal Formula Zuojin Pill in Suppressing Hepatocellular Carcinoma. Front. Pharmacol. 10, 1185. 10.3389/fphar.2019.01185 31649545 PMC6795061

[B13] Han J. Wang Z. Xing W. Yuan Y. Zhang Y. Lv T. (2017). Effect of Gegen Qinlian Decoction on Cardiac Gene Expression in Diabetic Mice. Int. J. Genomics 2017, 7421761. 10.1155/2017/7421761 29379793 PMC5742884

[B14] Haynes W. (2013). “Benjamini-Hochberg Method,” in Encyclopedia of Systems Biology. Editors Dubitzky W. Wolkenhauer O. Cho K.-H. Yokota H. (New York, NY: Springer New York), 78. 10.1007/978-1-4419-9863-7_1215

[B15] Heinrich M. Appendino G. Efferth T. Fürst R. Izzo A. A. Kayser O. (2020). Best Practice in Research - Overcoming Common Challenges in Phytopharmacological Research. J. Ethnopharmacology 246, 112230. 10.1016/j.jep.2019.112230 31526860

[B16] Huang D. Sherman B. T. Tan Q. Collins J. R. Alvord W. G. Roayaei J. (2007). The DAVID Gene Functional Classification Tool: a Novel Biological Module-Centric Algorithm to Functionally Analyze Large Gene Lists. Genome Biol. 8, R183. 10.1186/gb-2007-8-9-r183 17784955 PMC2375021

[B17] Huang X. Liu G. Guo J. Su Z. (2018). The PI3K/AKT Pathway in Obesity and Type 2 Diabetes. Int. J. Biol. Sci. 14, 1483–1496. 10.7150/ijbs.27173 30263000 PMC6158718

[B18] Huang J. Chen F. Zhong Z. Tan H. Y. Wang N. Liu Y. (2020a). Interpreting the Pharmacological Mechanisms of Huachansu Capsules on Hepatocellular Carcinoma Through Combining Network Pharmacology and Experimental Evaluation. Front. Pharmacol. 11, 414. 10.3389/fphar.2020.00414 32308626 PMC7145978

[B19] Huang X. Shi Y. Chen H. Le R. Gong X. Xu K. (2020b). Isoliquiritigenin Prevents Hyperglycemia-Induced Renal Injuries by Inhibiting Inflammation and Oxidative Stress via SIRT1-dependent Mechanism. Cell Death Dis 11, 1040. 10.1038/s41419-020-03260-9 33288747 PMC7721869

[B20] Kalra S. Jena B. N. Yeravdekar R. (2018). Emotional and Psychological Needs of People with Diabetes. Indian J. Endocrinol. Metab. 22, 696–704. 10.4103/ijem.ijem_189_17 30294583 PMC6166557

[B21] Khan S. Kamal M. A. (2019). Wogonin Alleviates Hyperglycemia Through Increased Glucose Entry into Cells Via AKT/GLUT4 Pathway. Curr. Pharm. Des. 25, 2602. 10.2174/1381612825666190722115410 31333118

[B22] Khan K. H. Wong M. Rihawi K. Bodla S. Morganstein D. Banerji U. (2016a). Hyperglycemia and Phosphatidylinositol 3-Kinase/Protein Kinase B/Mammalian Target of Rapamycin (PI3K/AKT/mTOR) Inhibitors in Phase I Trials: Incidence, Predictive Factors, and Management. Oncologist 21, 855. 10.1634/theoncologist.2015-0248 27151652 PMC4943382

[B23] Khan S. Zhang D. Zhang Y. Li M. Wang C. Zheng Z. a.-O. (2016b). Wogonin Attenuates Diabetic Cardiomyopathy through its Anti-inflammatory and Anti-oxidative Properties. Mol. Cell Endocrinol. 428. 101. 10.1016/j.mce.2016.03.025 27013352

[B24] Li S. Zhang B. (2013). Traditional Chinese Medicine Network Pharmacology: Theory, Methodology and Application. Chin. J. Nat. Medicines 11, 110–120. 10.1016/s1875-5364(13)60037-0 23787177

[B25] Li H. Zhao L. Zhang B. Jiang Y. Wang X. Guo Y. (2014). A Network Pharmacology Approach to Determine Active Compounds and Action Mechanisms of Ge-Gen-Qin-Lian Decoction for Treatment of Type 2 Diabetes. Evid. Based Complement. Alternat Med. 2014, 495840. 10.1155/2014/495840 24527048 PMC3914348

[B26] Li R. Chen Y. Shi M. Xu X. Zhao Y. Wu X. (2016). Gegen Qinlian Decoction Alleviates Experimental Colitis via Suppressing TLR4/NF-κB Signaling and Enhancing Antioxidant Effect. Phytomedicine 23, 1012–1020. 10.1016/j.phymed.2016.06.010 27444346

[B27] Li S. (2021). Network Pharmacology Evaluation Method Guidance - Draft. World J. Traditional Chin. Med. 7, 146–154. 10.4103/wjtcm.wjtcm_11_21

[B28] Loria P. Lonardo A. Anania F. (2013). Liver and Diabetes. A Vicious circle. Hepatol. Res. : official J. Jpn. Soc. Hepatol. 43, 51–64. 10.1111/j.1872-034x.2012.01031.x PMC3733501 23332087

[B29] Lucchesi A. N. Cassettari L. L. Spadella C. T. (2015). Alloxan-Induced Diabetes Causes Morphological and Ultrastructural Changes in Rat Liver that Resemble the Natural History of Chronic Fatty Liver Disease in Humans. J. Diabetes Res. 2015, 494578. 10.1155/2015/494578 25789328 PMC4350960

[B30] Lv J. Jia Y. Li J. Kuai W. Li Y. Guo F. (2019). Gegen Qinlian Decoction Enhances the Effect of PD-1 Blockade in Colorectal Cancer with Microsatellite Stability by Remodelling the Gut Microbiota and the Tumour Microenvironment. Cel Death Dis 10, 415. 10.1038/s41419-019-1638-6 PMC6538740 31138779

[B31] Pandey M. K. Kumar R. Pandey A. K. Soni P. Gangurde S. S. Sudini H. K. (2019). Mitigating Aflatoxin Contamination in Groundnut through A Combination of Genetic Resistance and Post-Harvest Management Practices. Toxins (Basel) 11, 315. 10.3390/toxins11060315 PMC6628460 31163657

[B32] Pang B. Zhou Q. Zhao T.-Y. He L.-S. Guo J. Chen H.-D. (2015). Innovative Thoughts on Treating Diabetes from the Perspective of Traditional Chinese Medicine. Evidence-Based Complement. Altern. Med. 2015, 905432. 10.1155/2015/905432 PMC4609429 26504482

[B33] Qiao X. Wang Q. Wang S. Miao W. J. Li Y. J. Xiang C. (2016). Compound to Extract to Formulation: a Knowledge-Transmitting Approach for Metabolites Identification of Gegen-Qinlian Decoction, a Traditional Chinese Medicine Formula. Sci. Rep. 6, 39534. 10.1038/srep39534 27996040 PMC5171860

[B34] Qiao X. Wang Q. Wang S. Kuang Y. Li K. Song W. (2018). A 42-Markers Pharmacokinetic Study Reveals Interactions of Berberine and Glycyrrhizic Acid in the Anti-diabetic Chinese Medicine Formula Gegen-Qinlian Decoction. Front. Pharmacol. 9, 622. 10.3389/fphar.2018.00622 29971002 PMC6018403

[B35] Reagan-Shaw S. Nihal M. Ahmad N. (2008). Dose Translation from Animal to Human Studies Revisited. FASEB J. 22, 659–661. 10.1096/fj.07-9574LSF 17942826

[B36] Ryuk J. A. Lixia M. Cao S. Ko B.-S. Park S. (2017). Efficacy and Safety of Gegen Qinlian Decoction for Normalizing Hyperglycemia in Diabetic Patients: A Systematic Review and Meta-Analysis of Randomized Clinical Trials. Complement. Therapies Med. 33, 6–13. 10.1016/j.ctim.2017.05.004 28735827

[B37] Shannon P. Markiel A. Ozier O. Baliga N. S. Wang J. T. Ramage D. (2003). Cytoscape: a Software Environment for Integrated Models of Biomolecular Interaction Networks. Genome Res. 13, 2498–2504. 10.1101/gr.1239303 14597658 PMC403769

[B38] Smith M. V. Lee M. J. Islam A. S. Rohrer J. L. Goldberg V. M. Beidelschies M. A. (2007). Inhibition of the PI3K-Akt Signaling Pathway Reduces Tumor Necrosis Factor-α Production in Response to Titanium Particles *In Vitro* . JBJS 89, 1019–1027. 10.2106/jbjs.f.00615 17473139

[B39] Srinivasan K. Viswanad B. Asrat L. Kaul C. L. Ramarao P. (2005). Combination of High-Fat Diet-Fed and Low-Dose Streptozotocin-Treated Rat: a Model for Type 2 Diabetes and Pharmacological Screening. Pharmacol. Res. 52. 313. 10.1016/j.phrs.2005.05.004 15979893

[B40] Tong X.-l. Zhao L.-h. Lian F.-m. Zhou Q. Xia L. Zhang J.-c. (2011). Clinical Observations on the Dose-Effect Relationship of Gegen Qin Lian Decoction () on 54 Out-Patients with Type 2 Diabetes. J. Traditional Chin. Med. 31, 56–59. 10.1016/s0254-6272(11)60013-7 21563509

[B41] Usuelli V. La Rocca E. (2015). Novel Therapeutic Approaches for Diabetic Nephropathy and Retinopathy. Pharmacol. Res. 98, 39–44. 10.1016/j.phrs.2014.10.003 25447794

[B42] Wang Q. Song W. Qiao X. Ji S. Kuang Y. Zhang Z. X. (2016). Simultaneous Quantification of 50 Bioactive Compounds of the Traditional Chinese Medicine Formula Gegen-Qinlian Decoction Using Ultra-high Performance Liquid Chromatography Coupled with Tandem Mass Spectrometry. J. Chromatogr. A. 1454, 15. 10.1016/j.chroma.2016.05.056 27262372

[B43] Wang J. Ran Q. Zeng H. R. Wang L. Hu C. J. Huang Q. W. (2018a). Cellular Stress Response Mechanisms of Rhizoma Coptidis: a Systematic Review. Chin. Med. 13, 27. 10.1186/s13020-018-0184-y 29930696 PMC5992750

[B44] Wang N. Yang B. Zhang X. Wang S. Zheng Y. Li X. (2018b). Network Pharmacology-Based Validation of Caveolin-1 as a Key Mediator of Ai Du Qing Inhibition of Drug Resistance in Breast Cancer. Front. Pharmacol. 9, 1106. 10.3389/fphar.2018.01106 30333750 PMC6176282

[B45] Wang Y. Zhang S. Li F. Zhou Y. Zhang Y. Wang Z. (2020). Therapeutic Target Database 2020: Enriched Resource for Facilitating Research and Early Development of Targeted Therapeutics. Nucleic Acids Res. 48, D1031, 10.1093/nar/gkz981 31691823 PMC7145558

[B46] Wu Y. Wang D. Yang X. Fu C. Zou L. Zhang J. (2019). Traditional Chinese Medicine Gegen Qinlian Decoction Ameliorates Irinotecan Chemotherapy-Induced Gut Toxicity in Mice. Biomed. Pharmacother. 109, 2252–2261. 10.1016/j.biopha.2018.11.095 30551482

[B47] Zeng Y. P. Huang Y. S. Hu Y. G. (2006). Effect of gegen qinlian decoction combined with short-term intensive insulin treatment on patients with type 2 diabetes mellitus of dampness-heat syndrome. Zhongguo Zhong Xi Yi Jie He Za Zhi 26, 514–520. 16841667

[B48] Zhang S. Wang L. Zan L. (2019). Investigation into the Underlying Molecular Mechanisms of white Adipose Tissue through Comparative Transcriptome Analysis of Multiple Tissues. Mol. Med. Rep. 19, 959–966. 10.3892/mmr.2018.9740 30569103 PMC6323223

[B49] Zheng Z. C. Zhu W. Lei L. Liu X. Q. Wu Y. G. (2020). Wogonin Ameliorates Renal Inflammation and Fibrosis by Inhibiting NF-κB and TGF-β1/Smad3 Signaling Pathways in Diabetic Nephropathy. Drug Des. Devel Ther. 14. 4135. 10.2147/DDDT.S274256 PMC7549498 33116403

[B50] Zhou J. Wang Q. Xiang Z. Tong Q. Pan J. Wan L. (2019). Network Pharmacology Analysis of Traditional Chinese Medicine Formula *Xiao Ke Yin Shui* Treating Type 2 Diabetes Mellitus. Evidence-Based Complement. Altern. Med. 2019, 4202563. 10.1155/2019/4202563 PMC6754917 31583009

[B51] Zhu X. Shi J. Li H. (2018). Liquiritigenin Attenuates High Glucose-Induced Mesangial Matrix Accumulation, Oxidative Stress, and Inflammation by Suppression of the NF-κB and NLRP3 Inflammasome Pathways. Biomed. Pharmacother. 106, 976, 10.1016/j.biopha.2018.07.045 30119269

