# Supplementary material for: Network Pharmacology–Based Analysis and Experimental Exploration of Antidiabetic Mechanisms of Gegen Qinlian Decoction
Source: Front Pharmacol. 2021 Jul 26;12:649606. doi: 10.3389/fphar.2021.649606 (PMC8350346; doi:10.3389/fphar.2021.649606)
Supplement: Supplementary file 1 [file DataSheet1.docx]

Supplementary Table S1 The chemical information of 42 compounds from GQL

| Number | Compounds | OB (%) | DL | Pubchem Cid | herbal name |
| --- | --- | --- | --- | --- | --- |
| MOL01 | Daidzin | 14.32 | 0.73 | 107971 | Gegen |
| MOL02 | 3-Methoxypuerarin | 11.73 | 0.78 | 5319485 | Gegen |
| MOL03 | Daidzein | 19.44 | 0.19 | 5281708 | Gegen |
| MOL04 | Genistin | 13.35 | 0.75 | 5281377 | Gegen |
| MOL05 | Formononetin 8-C-apiofuranosyl (1,6)glucoside | NA | NA | NA | Gegen |
| MOL06 | Genistein 8-C-apiofuranosyl(1,6)glucoside | NA | NA | NA | Gegen |
| MOL07 | Puerarin | 24.03 | 0.69 | 5281807 | Gegen |
| MOL08 | Wogonin | 30.68 | 0.23 | 5281703 | Huangqin |
| MOL09 | Oroxylin A | 41.37 | 0.23 | 5320315 | Huangqin |
| MOL10 | Chrysin 6-C-arabinoside-8-C-glucoside | NA | NA | 21722008 | Huangqin |
| MOL11 | Chrysin-8-C-arabinoside-6-C-glucoside | NA | NA | 133613134 | Huangqin |
| MOL12 | Baicalein | 33.52 | 0.21 | 5281605 | Huangqin |
| MOL13 | Wogonin 5-O-glucoside | NA | NA | 44258554 | Huangqin |
| MOL14 | Norwogonin 7-O-glucuronide | NA | NA | 44258552 | Huangqin |
| MOL15 | Oroxylin A 7-O-glucuronide | NA | NA | 14655552 | Huangqin |
| MOL16 | Wogonoside | 7.07 | 0.77 | 3084961 | Huangqin |
| MOL17 | Baicalin | 40.12 | 0.75 | 64982 | Huangqin |
| MOL18 | Chrysin | 22.61 | 0.18 | 5281607 | Huangqin |
| MOL19 | Magnoflorine | 0.48 | 0.55 | 73337 | Huanglian |
| MOL20 | Demethyleneberberine | NA | NA | 363209 | Huanglian |
| MOL21 | Coptisine | 30.67 | 0.86 | 72322 | Huanglian/Huangqin |
| MOL22 | Epiberberine | 43.09 | 0.78 | 160876 | Huanglian/Huangqin |
| MOL23 | Jatrorrhizine | 19.65 | 0.59 | 72323 | Huanglian/Huangqin |
| MOL24 | Berberine | 36.86 | 0.78 | 2353 | Huanglian |
| MOL25 | Palmatine | 64.6 | 0.65 | 19009 | Huanglian |
| MOL26 | Liquiritin | NA | NA | NA | Gancao |
| MOL27 | Isoliquiritin | 8.61 | 0.6 | 5318591 | Gancao |
| MOL28 | Isoliquiritigenin | 85.32 | 0.15 | 638278 | Gancao |
| MOL29 | Liquiritigenin | 32.76 | 0.18 | 114829 | Gancao |
| MOL30 | Glycycoumarin | 23.56 | 0.44 | 5317756 | Gancao |
| MOL31 | Glycyrol | 90.78 | 0.67 | 5320083 | Gancao |
| MOL32 | Formononetin | 69.67 | 0.21 | 46881075 | Gancao |
| MOL33 | Liquiritin apioside | 29.23 | 0.82 | NA | Gancao |
| MOL34 | Isoliquiritin apioside | NA | NA | NA | Gancao |
| MOL35 | Licorice-saponin G2 | 6.39 | 0.11 | 14891565 | Gancao |
| MOL36 | Glycyrrhizic acid | 19.62 | 0.11 | 14982 | Gancao |
| MOL37 | 3'-Methoxymirificin | NA | NA | 132580191 | Gegen |
| MOL38 | Lateriflorein 7-O-glucuronide | NA | NA | NA | Huangqin |
| MOL39 | Ononin | 11.52 | 0.78 | 442813 | Gancao/Gengen |
| MOL40 | Glycyrrhetinic acid | 22.05 | 0.74 | 10114 | Gancao |
| MOL41 | (4S)-Puerol B 2''-O-glucopyranoside | NA | NA | NA | Gegen |
| MOL42 | Chrysin 7-O-glucuronide | 18.28 | 0.69 | 15558426 | Huangqin |

Figure S1 The chemical structure of active compound in GQL





Supplementary Table S2 Compounds and related target genes for T2DM

| Number | Compound | Protein name | Gene Name | Data |
| --- | --- | --- | --- | --- |
| MOL01 | Daidzin | Acetylcholinesterase | ACHE | TCMSP |
| MOL01 | Daidzin | Aldehyde dehydrogenase, mitochondrial | ALDH2 | TCMSP/SEA |
| MOL01 | Daidzin | Androgen receptor | AR | TCMSP |
| MOL01 | Daidzin | Dipeptidyl peptidase IV | DPP4 | TCMSP |
| MOL01 | Daidzin | Estrogen receptor | ESR1 | TCMSP |
| MOL01 | Daidzin | Coagulation factor Xa | F10 | TCMSP |
| MOL01 | Daidzin | Thrombin | F2 | TCMSP |
| MOL01 | Daidzin | Fibroblast growth factor 2 | FGF2 | SEA |
| MOL01 | Daidzin | Glycogen synthase kinase-3 beta | GSK3B | TCMSP |
| MOL01 | Daidzin | Interleukin-2 | IL2 | SEA |
| MOL01 | Daidzin | Interleukin-6 | IL6 | SEA |
| MOL01 | Daidzin | Vascular endothelial growth factor receptor 2 | KDR | TCMSP |
| MOL01 | Daidzin | Amine oxidase [flavin-containing] A | MAOA | TCMSP |
| MOL01 | Daidzin | Peroxisome proliferator activated receptor gamma | PPARG | TCMSP |
| MOL01 | Daidzin | Prostaglandin G/H synthase 2 | PTGS2 | TCMSP |
| MOL01 | Daidzin | Sodium/glucose cotransporter 1 | SLC5A1 | SEA |
| MOL01 | Daidzin | Sodium/glucose cotransporter 2 | SLC5A2 | SEA |
| MOL01 | Daidzin | Solute carrier family 5 member 4 | SLC5A4 | SEA |
| MOL01 | Daidzin | Vascular endothelial growth factor A | VEGFA | SEA |
| MOL01 | Daidzin | Xanthine dehydrogenase/oxidase | XDH | SEA |
| MOL02 | 3-Methoxypuerarin | Aldehyde dehydrogenase, mitochondrial | ALDH2 | SEA |
| MOL02 | 3-Methoxypuerarin | Androgen receptor | AR | TCMSP |
| MOL02 | 3-Methoxypuerarin | Cytochrome P450 1B1 | CYP1B1 | SEA |
| MOL02 | 3-Methoxypuerarin | Interleukin-2 | IL2 | SEA |
| MOL02 | 3-Methoxypuerarin | Interleukin-6 | IL6 | SEA |
| MOL02 | 3-Methoxypuerarin | Proto-oncogene serine/threonine-protein kinase Pim-1 | PIM1 | TCMSP |
| MOL02 | 3-Methoxypuerarin | mRNA of Protein-tyrosine phosphatase, non-receptor type 1 | PTPN1 | TCMSP |
| MOL02 | 3-Methoxypuerarin | Sodium/glucose cotransporter 1 | SLC5A1 | SEA |
| MOL02 | 3-Methoxypuerarin | Sodium/glucose cotransporter 2 | SLC5A2 | SEA |
| MOL02 | 3-Methoxypuerarin | Solute carrier family 5 member 4 | SLC5A4 | SEA |
| Number | Compound | Protein name | Gene Name | Data |
| MOL01 | Daidzin | Acetylcholinesterase | ACHE | TCMSP |
| MOL01 | Daidzin | Aldehyde dehydrogenase, mitochondrial | ALDH3 | TCMSP/SEA |
| MOL01 | Daidzin | Androgen receptor | AR | TCMSP |
| MOL01 | Daidzin | Dipeptidyl peptidase IV | DPP5 | TCMSP |
| MOL01 | Daidzin | Estrogen receptor | ESR2 | TCMSP |
| MOL01 | Daidzin | Coagulation factor Xa | F6 | TCMSP |
| MOL01 | Daidzin | Thrombin | F14 | TCMSP |
| MOL01 | Daidzin | Fibroblast growth factor 3 | FGF3 | SEA |
| MOL01 | Daidzin | Glycogen synthase kinase-3 beta | GSK3B | TCMSP |
| MOL01 | Daidzin | Interleukin-10 | IL10 | SEA |
| MOL01 | Daidzin | Interleukin-14 | IL14 | SEA |
| MOL01 | Daidzin | Vascular endothelial growth factor receptor 3 | KDR | TCMSP |
| MOL01 | Daidzin | Amine oxidase [flavin-containing] A | MAOA | TCMSP |
| MOL01 | Daidzin | Peroxisome proliferator activated receptor gamma | PPARG | TCMSP |
| MOL01 | Daidzin | Prostaglandin G/H synthase 3 | PTGS3 | TCMSP |
| MOL01 | Daidzin | Sodium/glucose cotransporter 3 | SLC5A1 | SEA |
| MOL01 | Daidzin | Sodium/glucose cotransporter 4 | SLC5A2 | SEA |
| MOL01 | Daidzin | Solute carrier family 5 member 5 | SLC5A4 | SEA |
| MOL01 | Daidzin | Vascular endothelial growth factor A | VEGFA | SEA |
| MOL01 | Daidzin | Xanthine dehydrogenase/oxidase | XDH | SEA |
| MOL02 | 3-Methoxypuerarin | Aldehyde dehydrogenase, mitochondrial | ALDH3 | SEA |
| MOL02 | 3-Methoxypuerarin | Androgen receptor | AR | TCMSP |
| MOL02 | 3-Methoxypuerarin | Cytochrome P450 1B2 | CYP1B2 | SEA |
| MOL02 | 3-Methoxypuerarin | Interleukin-10 | IL10 | SEA |
| MOL02 | 3-Methoxypuerarin | Interleukin-14 | IL14 | SEA |
| MOL02 | 3-Methoxypuerarin | Proto-oncogene serine/threonine-protein kinase Pim-2 | PIM2 | TCMSP |
| MOL02 | 3-Methoxypuerarin | mRNA of Protein-tyrosine phosphatase, non-receptor type 2 | PTPN2 | TCMSP |
| MOL02 | 3-Methoxypuerarin | Sodium/glucose cotransporter 3 | SLC5A1 | SEA |
| MOL02 | 3-Methoxypuerarin | Sodium/glucose cotransporter 4 | SLC5A2 | SEA |
| MOL02 | 3-Methoxypuerarin | Solute carrier family 5 member 5 | SLC5A4 | SEA |
| Number | Compound | Protein name | Gene Name | Data |
| MOL01 | Daidzin | Acetylcholinesterase | ACHE | TCMSP |
| MOL01 | Daidzin | Aldehyde dehydrogenase, mitochondrial | ALDH4 | TCMSP/SEA |
| MOL01 | Daidzin | Androgen receptor | AR | TCMSP |
| MOL01 | Daidzin | Dipeptidyl peptidase IV | DPP6 | TCMSP |
| MOL01 | Daidzin | Estrogen receptor | ESR3 | TCMSP |
| MOL01 | Daidzin | Coagulation factor Xa | F22 | TCMSP |
| MOL01 | Daidzin | Thrombin | F30 | TCMSP |
| MOL01 | Daidzin | Fibroblast growth factor 4 | FGF4 | SEA |
| MOL01 | Daidzin | Glycogen synthase kinase-3 beta | GSK3B | TCMSP |
| MOL01 | Daidzin | Interleukin-18 | IL18 | SEA |
| MOL01 | Daidzin | Interleukin-22 | IL22 | SEA |
| MOL01 | Daidzin | Vascular endothelial growth factor receptor 4 | KDR | TCMSP |
| MOL01 | Daidzin | Amine oxidase [flavin-containing] A | MAOA | TCMSP |
| MOL01 | Daidzin | Peroxisome proliferator activated receptor gamma | PPARG | TCMSP |
| MOL01 | Daidzin | Prostaglandin G/H synthase 4 | PTGS4 | TCMSP |
| MOL01 | Daidzin | Sodium/glucose cotransporter 5 | SLC5A1 | SEA |
| MOL01 | Daidzin | Sodium/glucose cotransporter 6 | SLC5A2 | SEA |
| MOL01 | Daidzin | Solute carrier family 5 member 6 | SLC5A4 | SEA |
| MOL01 | Daidzin | Vascular endothelial growth factor A | VEGFA | SEA |
| MOL01 | Daidzin | Xanthine dehydrogenase/oxidase | XDH | SEA |
| MOL02 | 3-Methoxypuerarin | Aldehyde dehydrogenase, mitochondrial | ALDH4 | SEA |
| MOL02 | 3-Methoxypuerarin | Androgen receptor | AR | TCMSP |
| MOL02 | 3-Methoxypuerarin | Cytochrome P450 1B3 | CYP1B3 | SEA |
| MOL02 | 3-Methoxypuerarin | Interleukin-18 | IL18 | SEA |
| MOL02 | 3-Methoxypuerarin | Interleukin-22 | IL22 | SEA |
| MOL02 | 3-Methoxypuerarin | Proto-oncogene serine/threonine-protein kinase Pim-3 | PIM3 | TCMSP |
| MOL02 | 3-Methoxypuerarin | mRNA of Protein-tyrosine phosphatase, non-receptor type 3 | PTPN3 | TCMSP |
| MOL02 | 3-Methoxypuerarin | Sodium/glucose cotransporter 5 | SLC5A1 | SEA |
| MOL02 | 3-Methoxypuerarin | Sodium/glucose cotransporter 6 | SLC5A2 | SEA |
| MOL02 | 3-Methoxypuerarin | Solute carrier family 5 member 6 | SLC5A4 | SEA |
| Number | Compound | Protein name | Gene Name | Data |
| MOL01 | Daidzin | Acetylcholinesterase | ACHE | TCMSP |
| MOL01 | Daidzin | Aldehyde dehydrogenase, mitochondrial | ALDH5 | TCMSP/SEA |
| MOL01 | Daidzin | Androgen receptor | AR | TCMSP |
| MOL01 | Daidzin | Dipeptidyl peptidase IV | DPP7 | TCMSP |
| MOL01 | Daidzin | Estrogen receptor | ESR4 | TCMSP |
| MOL01 | Daidzin | Coagulation factor Xa | F38 | TCMSP |
| MOL01 | Daidzin | Thrombin | F46 | TCMSP |
| MOL01 | Daidzin | Fibroblast growth factor 5 | FGF5 | SEA |
| MOL01 | Daidzin | Glycogen synthase kinase-3 beta | GSK3B | TCMSP |
| MOL01 | Daidzin | Interleukin-26 | IL26 | SEA |
| MOL01 | Daidzin | Interleukin-30 | IL30 | SEA |
| MOL01 | Daidzin | Vascular endothelial growth factor receptor 5 | KDR | TCMSP |
| MOL01 | Daidzin | Amine oxidase [flavin-containing] A | MAOA | TCMSP |
| MOL01 | Daidzin | Peroxisome proliferator activated receptor gamma | PPARG | TCMSP |
| MOL01 | Daidzin | Prostaglandin G/H synthase 5 | PTGS5 | TCMSP |
| MOL01 | Daidzin | Sodium/glucose cotransporter 7 | SLC5A1 | SEA |
| MOL01 | Daidzin | Sodium/glucose cotransporter 8 | SLC5A2 | SEA |
| MOL01 | Daidzin | Solute carrier family 5 member 7 | SLC5A4 | SEA |
| MOL01 | Daidzin | Vascular endothelial growth factor A | VEGFA | SEA |
| MOL01 | Daidzin | Xanthine dehydrogenase/oxidase | XDH | SEA |
| MOL02 | 3-Methoxypuerarin | Aldehyde dehydrogenase, mitochondrial | ALDH5 | SEA |
| MOL02 | 3-Methoxypuerarin | Androgen receptor | AR | TCMSP |
| MOL02 | 3-Methoxypuerarin | Cytochrome P450 1B4 | CYP1B4 | SEA |
| MOL02 | 3-Methoxypuerarin | Interleukin-26 | IL26 | SEA |
| MOL02 | 3-Methoxypuerarin | Interleukin-30 | IL30 | SEA |
| MOL02 | 3-Methoxypuerarin | Proto-oncogene serine/threonine-protein kinase Pim-4 | PIM4 | TCMSP |
| MOL02 | 3-Methoxypuerarin | mRNA of Protein-tyrosine phosphatase, non-receptor type 4 | PTPN4 | TCMSP |
| MOL02 | 3-Methoxypuerarin | Sodium/glucose cotransporter 7 | SLC5A1 | SEA |
| MOL02 | 3-Methoxypuerarin | Sodium/glucose cotransporter 8 | SLC5A2 | SEA |
| MOL02 | 3-Methoxypuerarin | Solute carrier family 5 member 7 | SLC5A4 | SEA |
| Number | Compound | Protein name | Gene Name | Data |
| MOL01 | Daidzin | Acetylcholinesterase | ACHE | TCMSP |
| MOL01 | Daidzin | Aldehyde dehydrogenase, mitochondrial | ALDH6 | TCMSP/SEA |
| MOL01 | Daidzin | Androgen receptor | AR | TCMSP |
| MOL01 | Daidzin | Dipeptidyl peptidase IV | DPP8 | TCMSP |
| MOL01 | Daidzin | Estrogen receptor | ESR5 | TCMSP |
| MOL01 | Daidzin | Coagulation factor Xa | F54 | TCMSP |
| MOL01 | Daidzin | Thrombin | F62 | TCMSP |
| MOL01 | Daidzin | Fibroblast growth factor 6 | FGF6 | SEA |
| MOL01 | Daidzin | Glycogen synthase kinase-3 beta | GSK3B | TCMSP |
| MOL01 | Daidzin | Interleukin-34 | IL34 | SEA |
| MOL01 | Daidzin | Interleukin-38 | IL38 | SEA |
| MOL01 | Daidzin | Vascular endothelial growth factor receptor 6 | KDR | TCMSP |
| MOL01 | Daidzin | Amine oxidase [flavin-containing] A | MAOA | TCMSP |
| MOL01 | Daidzin | Peroxisome proliferator activated receptor gamma | PPARG | TCMSP |
| MOL01 | Daidzin | Prostaglandin G/H synthase 6 | PTGS6 | TCMSP |
| MOL01 | Daidzin | Sodium/glucose cotransporter 9 | SLC5A1 | SEA |
| MOL01 | Daidzin | Sodium/glucose cotransporter 10 | SLC5A2 | SEA |
| MOL01 | Daidzin | Solute carrier family 5 member 8 | SLC5A4 | SEA |
| MOL01 | Daidzin | Vascular endothelial growth factor A | VEGFA | SEA |
| MOL01 | Daidzin | Xanthine dehydrogenase/oxidase | XDH | SEA |
| MOL02 | 3-Methoxypuerarin | Aldehyde dehydrogenase, mitochondrial | ALDH6 | SEA |
| MOL02 | 3-Methoxypuerarin | Androgen receptor | AR | TCMSP |
| MOL02 | 3-Methoxypuerarin | Cytochrome P450 1B5 | CYP1B5 | SEA |
| MOL02 | 3-Methoxypuerarin | Interleukin-34 | IL34 | SEA |
| MOL02 | 3-Methoxypuerarin | Interleukin-38 | IL38 | SEA |
| MOL02 | 3-Methoxypuerarin | Proto-oncogene serine/threonine-protein kinase Pim-5 | PIM5 | TCMSP |
| MOL02 | 3-Methoxypuerarin | mRNA of Protein-tyrosine phosphatase, non-receptor type 5 | PTPN5 | TCMSP |
| MOL02 | 3-Methoxypuerarin | Sodium/glucose cotransporter 9 | SLC5A1 | SEA |
| MOL02 | 3-Methoxypuerarin | Sodium/glucose cotransporter 10 | SLC5A2 | SEA |
| MOL02 | 3-Methoxypuerarin | Solute carrier family 5 member 8 | SLC5A4 | SEA |
| Number | Compound | Protein name | Gene Name | Data |
| MOL01 | Daidzin | Acetylcholinesterase | ACHE | TCMSP |
| MOL01 | Daidzin | Aldehyde dehydrogenase, mitochondrial | ALDH7 | TCMSP/SEA |
| MOL01 | Daidzin | Androgen receptor | AR | TCMSP |
| MOL01 | Daidzin | Dipeptidyl peptidase IV | DPP9 | TCMSP |
| MOL01 | Daidzin | Estrogen receptor | ESR6 | TCMSP |
| MOL01 | Daidzin | Coagulation factor Xa | F70 | TCMSP |
| MOL01 | Daidzin | Thrombin | F78 | TCMSP |
| MOL01 | Daidzin | Fibroblast growth factor 7 | FGF7 | SEA |
| MOL01 | Daidzin | Glycogen synthase kinase-3 beta | GSK3B | TCMSP |
| MOL01 | Daidzin | Interleukin-42 | IL42 | SEA |
| MOL01 | Daidzin | Interleukin-46 | IL46 | SEA |
| MOL01 | Daidzin | Vascular endothelial growth factor receptor 7 | KDR | TCMSP |
| MOL01 | Daidzin | Amine oxidase [flavin-containing] A | MAOA | TCMSP |
| MOL01 | Daidzin | Peroxisome proliferator activated receptor gamma | PPARG | TCMSP |
| MOL01 | Daidzin | Prostaglandin G/H synthase 7 | PTGS7 | TCMSP |
| MOL01 | Daidzin | Sodium/glucose cotransporter 11 | SLC5A1 | SEA |
| MOL01 | Daidzin | Sodium/glucose cotransporter 12 | SLC5A2 | SEA |
| MOL01 | Daidzin | Solute carrier family 5 member 9 | SLC5A4 | SEA |
| MOL01 | Daidzin | Vascular endothelial growth factor A | VEGFA | SEA |
| MOL01 | Daidzin | Xanthine dehydrogenase/oxidase | XDH | SEA |
| MOL02 | 3-Methoxypuerarin | Aldehyde dehydrogenase, mitochondrial | ALDH7 | SEA |
| MOL02 | 3-Methoxypuerarin | Androgen receptor | AR | TCMSP |
| MOL02 | 3-Methoxypuerarin | Cytochrome P450 1B6 | CYP1B6 | SEA |
| MOL02 | 3-Methoxypuerarin | Interleukin-42 | IL42 | SEA |
| MOL02 | 3-Methoxypuerarin | Interleukin-46 | IL46 | SEA |
| MOL02 | 3-Methoxypuerarin | Proto-oncogene serine/threonine-protein kinase Pim-6 | PIM6 | TCMSP |
| MOL02 | 3-Methoxypuerarin | mRNA of Protein-tyrosine phosphatase, non-receptor type 6 | PTPN6 | TCMSP |
| MOL02 | 3-Methoxypuerarin | Sodium/glucose cotransporter 11 | SLC5A1 | SEA |
| MOL02 | 3-Methoxypuerarin | Sodium/glucose cotransporter 12 | SLC5A2 | SEA |
| MOL02 | 3-Methoxypuerarin | Solute carrier family 5 member 9 | SLC5A4 | SEA |
| Number | Compound | Protein name | Gene Name | Data |
| MOL01 | Daidzin | Acetylcholinesterase | ACHE | TCMSP |
| MOL01 | Daidzin | Aldehyde dehydrogenase, mitochondrial | ALDH8 | TCMSP/SEA |
| MOL01 | Daidzin | Androgen receptor | AR | TCMSP |
| MOL01 | Daidzin | Dipeptidyl peptidase IV | DPP10 | TCMSP |
| MOL01 | Daidzin | Estrogen receptor | ESR7 | TCMSP |
| MOL01 | Daidzin | Coagulation factor Xa | F86 | TCMSP |
| MOL01 | Daidzin | Thrombin | F94 | TCMSP |
| MOL01 | Daidzin | Fibroblast growth factor 8 | FGF8 | SEA |
| MOL01 | Daidzin | Glycogen synthase kinase-3 beta | GSK3B | TCMSP |
| MOL01 | Daidzin | Interleukin-50 | IL50 | SEA |
| MOL01 | Daidzin | Interleukin-54 | IL54 | SEA |
| MOL01 | Daidzin | Vascular endothelial growth factor receptor 8 | KDR | TCMSP |
| MOL01 | Daidzin | Amine oxidase [flavin-containing] A | MAOA | TCMSP |
| MOL01 | Daidzin | Peroxisome proliferator activated receptor gamma | PPARG | TCMSP |
| MOL01 | Daidzin | Prostaglandin G/H synthase 8 | PTGS8 | TCMSP |
| MOL01 | Daidzin | Sodium/glucose cotransporter 13 | SLC5A1 | SEA |
| MOL01 | Daidzin | Sodium/glucose cotransporter 14 | SLC5A2 | SEA |
| MOL01 | Daidzin | Solute carrier family 5 member 10 | SLC5A4 | SEA |
| MOL01 | Daidzin | Vascular endothelial growth factor A | VEGFA | SEA |
| MOL01 | Daidzin | Xanthine dehydrogenase/oxidase | XDH | SEA |
| MOL02 | 3-Methoxypuerarin | Aldehyde dehydrogenase, mitochondrial | ALDH8 | SEA |
| MOL02 | 3-Methoxypuerarin | Androgen receptor | AR | TCMSP |
| MOL02 | 3-Methoxypuerarin | Cytochrome P450 1B7 | CYP1B7 | SEA |
| MOL02 | 3-Methoxypuerarin | Interleukin-50 | IL50 | SEA |
| MOL02 | 3-Methoxypuerarin | Interleukin-54 | IL54 | SEA |
| MOL02 | 3-Methoxypuerarin | Proto-oncogene serine/threonine-protein kinase Pim-7 | PIM7 | TCMSP |
| MOL02 | 3-Methoxypuerarin | mRNA of Protein-tyrosine phosphatase, non-receptor type 7 | PTPN7 | TCMSP |
| MOL02 | 3-Methoxypuerarin | Sodium/glucose cotransporter 13 | SLC5A1 | SEA |
| MOL02 | 3-Methoxypuerarin | Sodium/glucose cotransporter 14 | SLC5A2 | SEA |
| MOL02 | 3-Methoxypuerarin | Solute carrier family 5 member 10 | SLC5A4 | SEA |
| Number | Compound | Protein name | Gene Name | Data |
| MOL01 | Daidzin | Acetylcholinesterase | ACHE | TCMSP |
| MOL01 | Daidzin | Aldehyde dehydrogenase, mitochondrial | ALDH9 | TCMSP/SEA |
| MOL01 | Daidzin | Androgen receptor | AR | TCMSP |
| MOL01 | Daidzin | Dipeptidyl peptidase IV | DPP11 | TCMSP |
| MOL01 | Daidzin | Estrogen receptor | ESR8 | TCMSP |
| MOL01 | Daidzin | Coagulation factor Xa | F102 | TCMSP |
| MOL01 | Daidzin | Thrombin | F110 | TCMSP |
| MOL01 | Daidzin | Fibroblast growth factor 9 | FGF9 | SEA |
| MOL01 | Daidzin | Glycogen synthase kinase-3 beta | GSK3B | TCMSP |
| MOL01 | Daidzin | Interleukin-58 | IL58 | SEA |
| MOL01 | Daidzin | Interleukin-62 | IL62 | SEA |
| MOL01 | Daidzin | Vascular endothelial growth factor receptor 9 | KDR | TCMSP |
| MOL01 | Daidzin | Amine oxidase [flavin-containing] A | MAOA | TCMSP |
| MOL01 | Daidzin | Peroxisome proliferator activated receptor gamma | PPARG | TCMSP |
| MOL01 | Daidzin | Prostaglandin G/H synthase 9 | PTGS9 | TCMSP |
| MOL01 | Daidzin | Sodium/glucose cotransporter 15 | SLC5A1 | SEA |
| MOL01 | Daidzin | Sodium/glucose cotransporter 16 | SLC5A2 | SEA |
| MOL01 | Daidzin | Solute carrier family 5 member 11 | SLC5A4 | SEA |
| MOL01 | Daidzin | Vascular endothelial growth factor A | VEGFA | SEA |
| MOL01 | Daidzin | Xanthine dehydrogenase/oxidase | XDH | SEA |
| MOL02 | 3-Methoxypuerarin | Aldehyde dehydrogenase, mitochondrial | ALDH9 | SEA |
| MOL02 | 3-Methoxypuerarin | Androgen receptor | AR | TCMSP |
| MOL02 | 3-Methoxypuerarin | Cytochrome P450 1B8 | CYP1B8 | SEA |
| MOL02 | 3-Methoxypuerarin | Interleukin-58 | IL58 | SEA |
| MOL02 | 3-Methoxypuerarin | Interleukin-62 | IL62 | SEA |
| MOL02 | 3-Methoxypuerarin | Proto-oncogene serine/threonine-protein kinase Pim-8 | PIM8 | TCMSP |
| MOL02 | 3-Methoxypuerarin | mRNA of Protein-tyrosine phosphatase, non-receptor type 8 | PTPN8 | TCMSP |
| MOL02 | 3-Methoxypuerarin | Sodium/glucose cotransporter 15 | SLC5A1 | SEA |
| MOL02 | 3-Methoxypuerarin | Sodium/glucose cotransporter 16 | SLC5A2 | SEA |
| MOL02 | 3-Methoxypuerarin | Solute carrier family 5 member 11 | SLC5A4 | SEA |
| Number | Compound | Protein name | Gene Name | Data |
| MOL01 | Daidzin | Acetylcholinesterase | ACHE | TCMSP |
| MOL01 | Daidzin | Aldehyde dehydrogenase, mitochondrial | ALDH10 | TCMSP/SEA |
| MOL01 | Daidzin | Androgen receptor | AR | TCMSP |
| MOL01 | Daidzin | Dipeptidyl peptidase IV | DPP12 | TCMSP |
| MOL01 | Daidzin | Estrogen receptor | ESR9 | TCMSP |
| MOL01 | Daidzin | Coagulation factor Xa | F118 | TCMSP |
| MOL01 | Daidzin | Thrombin | F126 | TCMSP |
| MOL01 | Daidzin | Fibroblast growth factor 10 | FGF10 | SEA |
| MOL01 | Daidzin | Glycogen synthase kinase-3 beta | GSK3B | TCMSP |
| MOL01 | Daidzin | Interleukin-66 | IL66 | SEA |
| MOL01 | Daidzin | Interleukin-70 | IL70 | SEA |
| MOL01 | Daidzin | Vascular endothelial growth factor receptor 10 | KDR | TCMSP |
| MOL01 | Daidzin | Amine oxidase [flavin-containing] A | MAOA | TCMSP |
| MOL01 | Daidzin | Peroxisome proliferator activated receptor gamma | PPARG | TCMSP |
| MOL01 | Daidzin | Prostaglandin G/H synthase 10 | PTGS10 | TCMSP |
| MOL01 | Daidzin | Sodium/glucose cotransporter 17 | SLC5A1 | SEA |
| MOL01 | Daidzin | Sodium/glucose cotransporter 18 | SLC5A2 | SEA |
| MOL01 | Daidzin | Solute carrier family 5 member 12 | SLC5A4 | SEA |
| MOL01 | Daidzin | Vascular endothelial growth factor A | VEGFA | SEA |
| MOL01 | Daidzin | Xanthine dehydrogenase/oxidase | XDH | SEA |
| MOL02 | 3-Methoxypuerarin | Aldehyde dehydrogenase, mitochondrial | ALDH10 | SEA |
| MOL02 | 3-Methoxypuerarin | Androgen receptor | AR | TCMSP |
| MOL02 | 3-Methoxypuerarin | Cytochrome P450 1B9 | CYP1B9 | SEA |
| MOL02 | 3-Methoxypuerarin | Interleukin-66 | IL66 | SEA |
| MOL02 | 3-Methoxypuerarin | Interleukin-70 | IL70 | SEA |
| MOL02 | 3-Methoxypuerarin | Proto-oncogene serine/threonine-protein kinase Pim-9 | PIM9 | TCMSP |
| MOL02 | 3-Methoxypuerarin | mRNA of Protein-tyrosine phosphatase, non-receptor type 9 | PTPN9 | TCMSP |
| MOL02 | 3-Methoxypuerarin | Sodium/glucose cotransporter 17 | SLC5A1 | SEA |
| MOL02 | 3-Methoxypuerarin | Sodium/glucose cotransporter 18 | SLC5A2 | SEA |
| MOL02 | 3-Methoxypuerarin | Solute carrier family 5 member 12 | SLC5A4 | SEA |
| Number | Compound | Protein name | Gene Name | Data |
| MOL01 | Daidzin | Acetylcholinesterase | ACHE | TCMSP |
| MOL01 | Daidzin | Aldehyde dehydrogenase, mitochondrial | ALDH11 | TCMSP/SEA |
| MOL01 | Daidzin | Androgen receptor | AR | TCMSP |
| MOL01 | Daidzin | Dipeptidyl peptidase IV | DPP13 | TCMSP |
| MOL01 | Daidzin | Estrogen receptor | ESR10 | TCMSP |
| MOL01 | Daidzin | Coagulation factor Xa | F134 | TCMSP |
| MOL01 | Daidzin | Thrombin | F142 | TCMSP |
| MOL01 | Daidzin | Fibroblast growth factor 11 | FGF11 | SEA |
| MOL01 | Daidzin | Glycogen synthase kinase-3 beta | GSK3B | TCMSP |
| MOL01 | Daidzin | Interleukin-74 | IL74 | SEA |
| MOL01 | Daidzin | Interleukin-78 | IL78 | SEA |
| MOL01 | Daidzin | Vascular endothelial growth factor receptor 11 | KDR | TCMSP |
| MOL01 | Daidzin | Amine oxidase [flavin-containing] A | MAOA | TCMSP |
| MOL01 | Daidzin | Peroxisome proliferator activated receptor gamma | PPARG | TCMSP |
| MOL01 | Daidzin | Prostaglandin G/H synthase 11 | PTGS11 | TCMSP |
| MOL01 | Daidzin | Sodium/glucose cotransporter 19 | SLC5A1 | SEA |
| MOL01 | Daidzin | Sodium/glucose cotransporter 20 | SLC5A2 | SEA |
| MOL01 | Daidzin | Solute carrier family 5 member 13 | SLC5A4 | SEA |
| MOL01 | Daidzin | Vascular endothelial growth factor A | VEGFA | SEA |
| MOL01 | Daidzin | Xanthine dehydrogenase/oxidase | XDH | SEA |
| MOL02 | 3-Methoxypuerarin | Aldehyde dehydrogenase, mitochondrial | ALDH11 | SEA |
| MOL02 | 3-Methoxypuerarin | Androgen receptor | AR | TCMSP |
| MOL02 | 3-Methoxypuerarin | Cytochrome P450 1B10 | CYP1B10 | SEA |
| MOL02 | 3-Methoxypuerarin | Interleukin-74 | IL74 | SEA |
| MOL02 | 3-Methoxypuerarin | Interleukin-78 | IL78 | SEA |
| MOL02 | 3-Methoxypuerarin | Proto-oncogene serine/threonine-protein kinase Pim-10 | PIM10 | TCMSP |
| MOL02 | 3-Methoxypuerarin | mRNA of Protein-tyrosine phosphatase, non-receptor type 10 | PTPN10 | TCMSP |
| MOL02 | 3-Methoxypuerarin | Sodium/glucose cotransporter 19 | SLC5A1 | SEA |
| MOL02 | 3-Methoxypuerarin | Sodium/glucose cotransporter 20 | SLC5A2 | SEA |
| MOL02 | 3-Methoxypuerarin | Solute carrier family 5 member 13 | SLC5A4 | SEA |
| Number | Compound | Protein name | Gene Name | Data |
| MOL01 | Daidzin | Acetylcholinesterase | ACHE | TCMSP |
| MOL01 | Daidzin | Aldehyde dehydrogenase, mitochondrial | ALDH12 | TCMSP/SEA |
| MOL01 | Daidzin | Androgen receptor | AR | TCMSP |
| MOL01 | Daidzin | Dipeptidyl peptidase IV | DPP14 | TCMSP |
| MOL01 | Daidzin | Estrogen receptor | ESR11 | TCMSP |
| MOL01 | Daidzin | Coagulation factor Xa | F150 | TCMSP |
| MOL01 | Daidzin | Thrombin | F158 | TCMSP |
| MOL01 | Daidzin | Fibroblast growth factor 12 | FGF12 | SEA |
| MOL01 | Daidzin | Glycogen synthase kinase-3 beta | GSK3B | TCMSP |
| MOL01 | Daidzin | Interleukin-82 | IL82 | SEA |
| MOL13 | Wogonin 5-O-glucoside | Sodium/glucose cotransporter 1 | SLC5A1 | SEA |
| MOL13 | Wogonin 5-O-glucoside | Sodium/glucose cotransporter 2 | SLC5A2 | SEA |
| MOL13 | Wogonin 5-O-glucoside | Solute carrier family 5 member 4 | SLC5A4 | SEA |
| MOL13 | Wogonin 5-O-glucoside | Vascular endothelial growth factor A | VEGFA | SEA |
| MOL13 | Wogonin 5-O-glucoside | Xanthine dehydrogenase/oxidase | XDH | SEA |
| MOL14 | Norwogonin 7-O-glucuronide | Multidrug resistance protein 1 | ABCB1 | SEA |
| MOL14 | Norwogonin 7-O-glucuronide | ATP-binding cassette sub-family G member 2 | ABCG2 | SEA |
| MOL14 | Norwogonin 7-O-glucuronide | Aldose reductase | AKR1B1 | SEA |
| MOL14 | Norwogonin 7-O-glucuronide | Aldo-keto reductase family 1 member B10 | AKR1B10 | SEA |
| MOL14 | Norwogonin 7-O-glucuronide | Cyclin-A2 | CCNA2 | SEA |
| MOL14 | Norwogonin 7-O-glucuronide | Cyclic AMP-responsive element-binding protein 1 | CREB1 | SEA |
| MOL14 | Norwogonin 7-O-glucuronide | Cytochrome P450 1A1 | CYP1A1 | SEA |
| MOL14 | Norwogonin 7-O-glucuronide | Cytochrome P450 1B1 | CYP1B1 | SEA |
| MOL14 | Norwogonin 7-O-glucuronide | Interleukin-2 | IL2 | SEA |
| MOL14 | Norwogonin 7-O-glucuronide | Placenta growth factor | PGF | SEA |
| MOL14 | Norwogonin 7-O-glucuronide | Vascular endothelial growth factor A | VEGFA | SEA |
| MOL14 | Norwogonin 7-O-glucuronide | Xanthine dehydrogenase/oxidase | XDH | SEA |
| MOL15 | Oroxylin A 7-O-glucuronide | Multidrug resistance protein 1 | ABCB1 | SEA |
| MOL15 | Oroxylin A 7-O-glucuronide | Multidrug resistance-associated protein 1 | ABCC1 | SEA |
| MOL15 | Oroxylin A 7-O-glucuronide | ATP-binding cassette sub-family G member 2 | ABCG2 | SEA |
| MOL15 | Oroxylin A 7-O-glucuronide | Aldose reductase | AKR1B1 | SEA |
| MOL15 | Oroxylin A 7-O-glucuronide | Aldo-keto reductase family 1 member B10 | AKR1B10 | SEA |
| MOL15 | Oroxylin A 7-O-glucuronide | Cyclic AMP-responsive element-binding protein 1 | CREB1 | SEA |
| MOL15 | Oroxylin A 7-O-glucuronide | Cytochrome P450 1A1 | CYP1A1 | SEA |
| MOL15 | Oroxylin A 7-O-glucuronide | Cytochrome P450 1B1 | CYP1B1 | SEA |
| MOL15 | Oroxylin A 7-O-glucuronide | Interleukin-2 | IL2 | SEA |
| MOL15 | Oroxylin A 7-O-glucuronide | NADPH oxidase 4 | NOX4 | SEA |
| MOL15 | Oroxylin A 7-O-glucuronide | Solute carrier family 5 member 4 | SLC5A4 | SEA |
| MOL15 | Oroxylin A 7-O-glucuronide | Xanthine dehydrogenase/oxidase | XDH | SEA |
| MOL16 | Wogonoside | Multidrug resistance protein 1 | ABCB1 | SEA |
| MOL16 | Wogonoside | Multidrug resistance-associated protein 1 | ABCC1 | SEA |
| MOL16 | Wogonoside | ATP-binding cassette sub-family G member 2 | ABCG2 | SEA |
| MOL16 | Wogonoside | Aldose reductase | AKR1B1 | SEA |
| MOL16 | Wogonoside | Cyclin-A2 | CCNA2 | SEA |
| MOL16 | Wogonoside | Cyclic AMP-responsive element-binding protein 1 | CREB1 | SEA |
| MOL16 | Wogonoside | Cytochrome P450 1A1 | CYP1A1 | SEA |
| MOL16 | Wogonoside | Cytochrome P450 1B1 | CYP1B1 | SEA |
| MOL16 | Wogonoside | Coagulation factor Xa | F10 | TCMSP |
| MOL16 | Wogonoside | Thrombin | F2 | TCMSP |
| MOL16 | Wogonoside | Interleukin-2 | IL2 | SEA |
| MOL16 | Wogonoside | Placenta growth factor | PGF | SEA |
| MOL16 | Wogonoside | mRNA of Protein-tyrosine phosphatase, non-receptor type 1 | PTPN1 | TCMSP |
| MOL16 | Wogonoside | Solute carrier family 5 member 4 | SLC5A4 | SEA |
| MOL16 | Wogonoside | Xanthine dehydrogenase/oxidase | XDH | SEA |
| MOL17 | Baicalin | Multidrug resistance protein 1 | ABCB1 | SEA |
| MOL17 | Baicalin | ATP-binding cassette sub-family G member 2 | ABCG2 | SEA |
| MOL17 | Baicalin | Aldose reductase | AKR1B1 | SEA |
| MOL17 | Baicalin | Aldo-keto reductase family 1 member B10 | AKR1B10 | SEA |
| MOL17 | Baicalin | Cyclic AMP-responsive element-binding protein 1 | CREB1 | SEA |
| MOL17 | Baicalin | Cytochrome P450 1A1 | CYP1A1 | SEA |
| MOL17 | Baicalin | Cytochrome P450 1B1 | CYP1B1 | SEA |
| MOL17 | Baicalin | Coagulation factor Xa | F10 | TCMSP |
| MOL17 | Baicalin | Interleukin-2 | IL2 | SEA |
| MOL17 | Baicalin | NADPH oxidase 4 | NOX4 | SEA |
| MOL17 | Baicalin | Nuclear receptor subfamily 4 group A member 2 | NR4A2 | SEA |
| MOL17 | Baicalin | Placenta growth factor | PGF | SEA |
| MOL17 | Baicalin | mRNA of Protein-tyrosine phosphatase, non-receptor type 1 | PTPN1 | TCMSP |
| MOL17 | Baicalin | Xanthine dehydrogenase/oxidase | XDH | SEA |
| MOL18 | Chrysin | Multidrug resistance protein 1 | ABCB1 | SEA |
| MOL18 | Chrysin | Multidrug resistance-associated protein 1 | ABCC1 | SEA |
| MOL18 | Chrysin | ATP-binding cassette sub-family G member 2 | ABCG2 | SEA |
| MOL18 | Chrysin | Aryl hydrocarbon receptor | AHR | SEA |
| MOL18 | Chrysin | Aldose reductase | AKR1B1 | SEA |
| MOL18 | Chrysin | Aldo-keto reductase family 1 member B10 | AKR1B10 | SEA |
| MOL18 | Chrysin | Aldehyde dehydrogenase, mitochondrial | ALDH2 | SEA |
| MOL18 | Chrysin | Amyloid-beta A4 protein | APP | SEA |
| MOL18 | Chrysin | Androgen receptor | AR | TCMSP/SEA |
| MOL18 | Chrysin | Cyclin-A2 | CCNA2 | SEA |
| MOL18 | Chrysin | Cyclin-dependent kinase inhibitor 1 | CDKN1B | TCMSP |
| MOL18 | Chrysin | Cyclic AMP-responsive element-binding protein 1 | CREB1 | SEA |
| MOL18 | Chrysin | Cytochrome P450 1A1 | CYP1A1 | SEA |
| MOL18 | Chrysin | Cytochrome P450 1A2 | CYP1A2 | SEA |
| MOL18 | Chrysin | Cytochrome P450 1B1 | CYP1B1 | SEA |
| MOL18 | Chrysin | Dipeptidyl peptidase IV | DPP4 | TCMSP |
| MOL18 | Chrysin | Epidermal growth factor receptor | EGFR | SEA |
| MOL18 | Chrysin | Estrogen receptor | ESR1 | SEA |
| MOL18 | Chrysin | Estrogen receptor beta | ESR2 | SEA |
| MOL18 | Chrysin | Steroid hormone receptor ERR1 | ESRRA | SEA |
| MOL18 | Chrysin | Thrombin | F2 | TCMSP |
| MOL18 | Chrysin | Glutathione S-transferase A1 | GSTA1 | SEA |
| MOL18 | Chrysin | Heat shock protein HSP 90 | HSP90AA1 | TCMSP |
| MOL18 | Chrysin | Interleukin-13 | IL13 | TCMSP |
| MOL18 | Chrysin | Interleukin-2 | IL2 | SEA |
| MOL18 | Chrysin | Interleukin-4 | IL4 | TCMSP |
| MOL18 | Chrysin | Amine oxidase [flavin-containing] A | MAOA | SEA |
| MOL18 | Chrysin | Myeloperoxidase | MPO | SEA |
| MOL18 | Chrysin | NADPH oxidase 4 | NOX4 | SEA |
| MOL18 | Chrysin | NAD(P)H dehydrogenase [quinone] 1 | NQO1 | SEA |
| MOL18 | Chrysin | Placenta growth factor | PGF | SEA |
| MOL18 | Chrysin | Phosphatidylinositol-4,5-bisphosphate 3-kinase catalytic subunit, gamma isoform | PIK3CG | TCMSP |
| MOL18 | Chrysin | Prostaglandin G/H synthase 1 | PTGS1 | TCMSP |
| MOL18 | Chrysin | Prostaglandin G/H synthase 2 | PTGS2 | TCMSP |
| MOL18 | Chrysin | Transforming growth factor beta-1 | TGFB1 | TCMSP |
| MOL18 | Chrysin | Transthyretin | TTR | SEA |
| MOL18 | Chrysin | Vascular endothelial growth factor A | VEGFA | SEA |
| MOL18 | Chrysin | Xanthine dehydrogenase/oxidase | XDH | SEA |
| MOL19 | Magnoflorine | Acetylcholinesterase | ACHE | TCMSP |
| MOL19 | Magnoflorine | Androgen receptor | AR | TCMSP |
| MOL19 | Magnoflorine | D(1A) dopamine receptor | DRD1 | SEA |
| MOL19 | Magnoflorine | Tissue factor | F3 | SEA |
| MOL19 | Magnoflorine | Heat shock protein HSP 90 | HSP90AA1 | TCMSP |
| MOL19 | Magnoflorine | Nitric-oxide synthase, endothelial | NOS3 | TCMSP |
| MOL19 | Magnoflorine | Pregnane X receptor | NR1I2 | TCMSP |
| MOL19 | Magnoflorine | Prostaglandin G/H synthase 1 | PTGS1 | TCMSP |
| MOL19 | Magnoflorine | Prostaglandin G/H synthase 2 | PTGS2 | TCMSP |
| MOL19 | Magnoflorine | Retinoic acid receptor RXR-alpha | RXRA | TCMSP |
| MOL20 | Demethyleneberberine | Cytochrome P450 1B1 | CYP1B1 | SEA |
| MOL20 | Demethyleneberberine | D(1A) dopamine receptor | DRD1 | SEA |
| MOL20 | Demethyleneberberine | Ras-related C3 botulinum toxin substrate 1 | RAC1 | SEA |
| MOL21 | Coptisine | Androgen receptor | AR | TCMSP |
| MOL21 | Coptisine | Cytochrome P450 1B1 | CYP1B1 | SEA |
| MOL21 | Coptisine | Estrogen receptor | ESR1 | TCMSP |
| MOL21 | Coptisine | Nitric oxide synthase, inducible | NOS2 | TCMSP |
| MOL21 | Coptisine | Nitric-oxide synthase, endothelial | NOS3 | TCMSP |
| MOL21 | Coptisine | Prostaglandin G/H synthase 1 | PTGS1 | TCMSP |
| MOL21 | Coptisine | Prostaglandin G/H synthase 2 | PTGS2 | TCMSP |
| MOL21 | Coptisine | Ras-related C3 botulinum toxin substrate 1 | RAC1 | SEA |
| MOL22 | Epiberberine | Androgen receptor | AR | TCMSP |
| MOL22 | Epiberberine | Bcl2-associated agonist of cell death | BAD | SEA |
| MOL22 | Epiberberine | Cytochrome P450 1B1 | CYP1B1 | SEA |
| MOL22 | Epiberberine | Estrogen receptor | ESR1 | TCMSP |
| MOL22 | Epiberberine | Nitric oxide synthase, inducible | NOS2 | TCMSP |
| MOL22 | Epiberberine | Nitric-oxide synthase, endothelial | NOS3 | TCMSP |
| MOL22 | Epiberberine | Prostaglandin G/H synthase 2 | PTGS2 | TCMSP |
| MOL22 | Epiberberine | Ras-related C3 botulinum toxin substrate 1 | RAC1 | SEA |
| MOL22 | Epiberberine | Retinoic acid receptor RXR-alpha | RXRA | TCMSP |
| MOL23 | Jatrorrhizine | Androgen receptor | AR | TCMSP |
| MOL23 | Jatrorrhizine | Cyclin-A2 | CCNA2 | TCMSP |
| MOL23 | Jatrorrhizine | Cell division protein kinase 2 | CDK2 | TCMSP |
| MOL23 | Jatrorrhizine | Cytochrome P450 1B1 | CYP1B1 | SEA |
| MOL23 | Jatrorrhizine | D(1A) dopamine receptor | DRD1 | SEA |
| MOL23 | Jatrorrhizine | Estrogen receptor | ESR1 | TCMSP |
| MOL23 | Jatrorrhizine | Estrogen receptor beta | ESR2 | TCMSP |
| MOL23 | Jatrorrhizine | Heat shock protein HSP 90 | HSP90AA1 | TCMSP |
| MOL23 | Jatrorrhizine | Nitric oxide synthase, inducible | NOS2 | TCMSP |
| MOL23 | Jatrorrhizine | Nitric-oxide synthase, endothelial | NOS3 | TCMSP |
| MOL23 | Jatrorrhizine | Proto-oncogene serine/threonine-protein kinase Pim-1 | PIM1 | TCMSP |
| MOL23 | Jatrorrhizine | Prostaglandin G/H synthase 1 | PTGS1 | TCMSP |
| MOL23 | Jatrorrhizine | Prostaglandin G/H synthase 2 | PTGS2 | TCMSP |
| MOL23 | Jatrorrhizine | Ras-related C3 botulinum toxin substrate 1 | RAC1 | SEA |
| MOL23 | Jatrorrhizine | Retinoic acid receptor RXR-alpha | RXRA | TCMSP |
| MOL24 | Berberine | Acetylcholinesterase | ACHE | SEA |
| MOL24 | Berberine | Androgen receptor | AR | TCMSP |
| MOL24 | Berberine | Cytochrome P450 1A2 | CYP1A2 | SEA |
| MOL24 | Berberine | Cytochrome P450 1B1 | CYP1B1 | SEA |
| MOL24 | Berberine | Estrogen receptor | ESR1 | TCMSP |
| MOL24 | Berberine | Coagulation factor Xa | F10 | TCMSP |
| MOL24 | Berberine | Heat shock protein HSP 90 | HSP90AA1 | TCMSP |
| MOL24 | Berberine | Nitric oxide synthase, inducible | NOS2 | TCMSP |
| MOL24 | Berberine | Nitric-oxide synthase, endothelial | NOS3 | TCMSP |
| MOL24 | Berberine | Prostaglandin G/H synthase 1 | PTGS1 | TCMSP |
| MOL24 | Berberine | Prostaglandin G/H synthase 2 | PTGS2 | TCMSP |
| MOL24 | Berberine | Ras-related C3 botulinum toxin substrate 1 | RAC1 | SEA |
| MOL24 | Berberine | Retinoic acid receptor RXR-alpha | RXRA | TCMSP |
| MOL25 | Palmatine | Acetylcholinesterase | ACHE | SEA |
| MOL25 | Palmatine | Androgen receptor | AR | TCMSP |
| MOL25 | Palmatine | Cell division protein kinase 2 | CDK2 | TCMSP |
| MOL25 | Palmatine | Cytochrome P450 1B1 | CYP1B1 | SEA |
| MOL25 | Palmatine | Estrogen receptor | ESR1 | TCMSP |
| MOL25 | Palmatine | Estrogen receptor beta | ESR2 | TCMSP |
| MOL25 | Palmatine | Heat shock protein HSP 90 | HSP90AA1 | TCMSP |
| MOL25 | Palmatine | Nitric oxide synthase, inducible | NOS2 | TCMSP |
| MOL25 | Palmatine | Nitric-oxide synthase, endothelial | NOS3 | TCMSP |
| MOL25 | Palmatine | Proto-oncogene serine/threonine-protein kinase Pim-1 | PIM1 | TCMSP |
| MOL25 | Palmatine | Prostaglandin G/H synthase 1 | PTGS1 | TCMSP |
| MOL25 | Palmatine | Prostaglandin G/H synthase 2 | PTGS2 | TCMSP |
| MOL25 | Palmatine | Ras-related C3 botulinum toxin substrate 1 | RAC1 | SEA |
| MOL25 | Palmatine | Retinoic acid receptor RXR-alpha | RXRA | TCMSP |
| MOL26 | Liquiritin | Cytochrome P450 1B1 | CYP1B1 | SEA |
| MOL26 | Liquiritin | Estrogen receptor | ESR1 | SEA |
| MOL26 | Liquiritin | Estrogen receptor beta | ESR2 | SEA |
| MOL26 | Liquiritin | Fibroblast growth factor 2 | FGF2 | SEA |
| MOL26 | Liquiritin | Interleukin-2 | IL2 | SEA |
| MOL26 | Liquiritin | Interleukin-6 | IL6 | SEA |
| MOL26 | Liquiritin | Sodium/glucose cotransporter 1 | SLC5A1 | SEA |
| MOL26 | Liquiritin | Sodium/glucose cotransporter 2 | SLC5A2 | SEA |
| MOL26 | Liquiritin | Solute carrier family 5 member 4 | SLC5A4 | SEA |
| MOL26 | Liquiritin | Vascular endothelial growth factor A | VEGFA | SEA |
| MOL26 | Liquiritin | Xanthine dehydrogenase/oxidase | XDH | SEA |
| MOL27 | Isoliquiritin | ATP-binding cassette sub-family G member 2 | ABCG2 | SEA |
| MOL27 | Isoliquiritin | Aldose reductase | AKR1B1 | SEA |
| MOL27 | Isoliquiritin | Aldo-keto reductase family 1 member B10 | AKR1B10 | SEA |
| MOL27 | Isoliquiritin | Beta-secretase 1 | BACE1 | SEA |
| MOL27 | Isoliquiritin | Tissue factor | F3 | SEA |
| MOL27 | Isoliquiritin | Fibroblast growth factor 2 | FGF2 | SEA |
| MOL27 | Isoliquiritin | Hexokinase-2 | HK2 | SEA |
| MOL27 | Isoliquiritin | Interleukin-2 | IL2 | SEA |
| MOL27 | Isoliquiritin | Microtubule-associated protein tau | MAPT | SEA |
| MOL27 | Isoliquiritin | [Pyruvate dehydrogenase (acetyl-transferring)] kinase isozyme 4, mitochondrial | PDK4 | SEA |
| MOL27 | Isoliquiritin | Sodium/glucose cotransporter 1 | SLC5A1 | SEA |
| MOL27 | Isoliquiritin | Sodium/glucose cotransporter 2 | SLC5A2 | SEA |
| MOL27 | Isoliquiritin | Solute carrier family 5 member 4 | SLC5A4 | SEA |
| MOL27 | Isoliquiritin | Vascular endothelial growth factor A | VEGFA | SEA |
| MOL27 | Isoliquiritin | Xanthine dehydrogenase/oxidase | XDH | SEA |
| MOL28 | Isoliquiritigenin | ATP-binding cassette sub-family G member 2 | ABCG2 | SEA |
| MOL28 | Isoliquiritigenin | Aldose reductase | AKR1B1 | SEA |
| MOL28 | Isoliquiritigenin | Aldo-keto reductase family 1 member B10 | AKR1B10 | SEA |
| MOL28 | Isoliquiritigenin | Amyloid-beta A4 protein | APP | SEA |
| MOL28 | Isoliquiritigenin | Androgen receptor | AR | TCMSP |
| MOL28 | Isoliquiritigenin | Beta-secretase 1 | BACE1 | SEA |
| MOL28 | Isoliquiritigenin | Apoptosis regulator BAX | BAX | TCMSP |
| MOL28 | Isoliquiritigenin | Cyclin-A2 | CCNA2 | TCMSP |
| MOL28 | Isoliquiritigenin | Cell division protein kinase 2 | CDK2 | TCMSP |
| MOL28 | Isoliquiritigenin | Cytochrome P450 1A1 | CYP1A1 | SEA |
| MOL28 | Isoliquiritigenin | Cytochrome P450 1A2 | CYP1A2 | SEA |
| MOL28 | Isoliquiritigenin | Cytochrome P450 1B1 | CYP1B1 | SEA |
| MOL28 | Isoliquiritigenin | Cytochrome P450 3A4 | CYP3A4 | SEA |
| MOL28 | Isoliquiritigenin | Estrogen receptor | ESR1 | TCMSP/SEA |
| MOL28 | Isoliquiritigenin | Estrogen receptor beta | ESR2 | TCMSP/SEA |
| MOL28 | Isoliquiritigenin | Tissue factor | F3 | SEA |
| MOL28 | Isoliquiritigenin | Proto-oncogene c-Fos | FOS | TCMSP/SEA |
| MOL28 | Isoliquiritigenin | Glycogen synthase kinase-3 beta | GSK3B | TCMSP |
| MOL28 | Isoliquiritigenin | Histone deacetylase 1 | HDAC1 | SEA |
| MOL28 | Isoliquiritigenin | Hypoxia-inducible factor 1-alpha | HIF1A | SEA |
| MOL28 | Isoliquiritigenin | Heat shock protein HSP 90 | HSP90AA1 | TCMSP/SEA |
| MOL28 | Isoliquiritigenin | 60 kDa heat shock protein, mitochondrial | HSPD1 | SEA |
| MOL28 | Isoliquiritigenin | Tyrosine-protein kinase JAK2 | JAK2 | TCMSP |
| MOL28 | Isoliquiritigenin | Transcription factor AP-1 | JUN | SEA |
| MOL28 | Isoliquiritigenin | Amine oxidase [flavin-containing] A | MAOA | SEA |
| MOL28 | Isoliquiritigenin | Mitogen-activated protein kinase 14 | MAPK14 | TCMSP |
| MOL28 | Isoliquiritigenin | Microtubule-associated protein tau | MAPT | SEA |
| MOL28 | Isoliquiritigenin | Interstitial collagenase | MMP1 | SEA |
| MOL28 | Isoliquiritigenin | 72 kDa type IV collagenase | MMP2 | SEA |
| MOL28 | Isoliquiritigenin | Matrix metalloproteinase-9 | MMP9 | SEA |
| MOL28 | Isoliquiritigenin | Metallothionein-2 | MT2A | TCMSP |
| MOL28 | Isoliquiritigenin | Nuclear factor erythroid 2-related factor 2 | NFE2L2 | SEA |
| MOL28 | Isoliquiritigenin | Nuclear factor NF-kappa-B p105 subunit | NFKB1 | SEA |
| MOL28 | Isoliquiritigenin | Nitric oxide synthase, inducible | NOS2 | TCMSP |
| MOL28 | Isoliquiritigenin | Nuclear receptor subfamily 0 group B member 2 | NR0B2 | SEA |
| MOL28 | Isoliquiritigenin | [Pyruvate dehydrogenase (acetyl-transferring)] kinase isozyme 1, mitochondrial | PDK1 | SEA |
| MOL28 | Isoliquiritigenin | [Pyruvate dehydrogenase (acetyl-transferring)] kinase isozyme 4, mitochondrial | PDK4 | SEA |
| MOL28 | Isoliquiritigenin | Phosphatidylinositol-4,5-bisphosphate 3-kinase catalytic subunit, gamma isoform | PIK3CG | TCMSP |
| MOL28 | Isoliquiritigenin | Proto-oncogene serine/threonine-protein kinase Pim-1 | PIM1 | TCMSP |
| MOL28 | Isoliquiritigenin | Peroxisome proliferator activated receptor gamma | PPARG | TCMSP |
| MOL28 | Isoliquiritigenin | Prostaglandin G/H synthase 1 | PTGS1 | TCMSP |
| MOL28 | Isoliquiritigenin | Prostaglandin G/H synthase 2 | PTGS2 | TCMSP |
| MOL28 | Isoliquiritigenin | E-selectin | SELE | TCMSP |
| MOL28 | Isoliquiritigenin | Solute carrier family 2, facilitated glucose transporter member 1 | SLC2A1 | TCMSP |
| MOL28 | Isoliquiritigenin | Tumor necrosis factor receptor superfamily member 1A | TNFRSF1A | SEA |
| MOL28 | Isoliquiritigenin | Transthyretin | TTR | SEA |
| MOL28 | Isoliquiritigenin | Vascular cell adhesion protein 1 | VCAM1 | TCMSP |
| MOL29 | Liquiritigenin | Cytochrome P450 1B1 | CYP1B1 | SEA |
| MOL29 | Liquiritigenin | Estrogen receptor | ESR1 | TCMSP/SEA |
| MOL29 | Liquiritigenin | Estrogen receptor beta | ESR2 | SEA |
| MOL29 | Liquiritigenin | Steroid hormone receptor ERR1 | ESRRA | SEA |
| MOL29 | Liquiritigenin | Heat shock protein HSP 90 | HSP90AA1 | TCMSP |
| MOL29 | Liquiritigenin | Placenta growth factor | PGF | SEA |
| MOL29 | Liquiritigenin | Phosphatidylinositol-4,5-bisphosphate 3-kinase catalytic subunit, gamma isoform | PIK3CG | TCMSP |
| MOL29 | Liquiritigenin | Prostaglandin G/H synthase 1 | PTGS1 | TCMSP |
| MOL29 | Liquiritigenin | Prostaglandin G/H synthase 2 | PTGS2 | TCMSP |
| MOL29 | Liquiritigenin | Retinoic acid receptor RXR-alpha | RXRA | TCMSP |
| MOL29 | Liquiritigenin | Vascular endothelial growth factor A | VEGFA | SEA |
| MOL30 | Glycycoumarin | ATP-binding cassette sub-family G member 2 | ABCG2 | SEA |
| MOL30 | Glycycoumarin | Cyclic AMP-responsive element-binding protein 1 | CREB1 | SEA |
| MOL30 | Glycycoumarin | Cytochrome P450 1B1 | CYP1B1 | SEA |
| MOL30 | Glycycoumarin | Estrogen receptor | ESR1 | SEA |
| MOL30 | Glycycoumarin | Estrogen receptor beta | ESR2 | SEA |
| MOL30 | Glycycoumarin | Hypoxia-inducible factor 1-alpha | HIF1A | SEA |
| MOL30 | Glycycoumarin | NAD(P)H dehydrogenase [quinone] 1 | NQO1 | SEA |
| MOL30 | Glycycoumarin | Serum paraoxonase/arylesterase 1 | PON1 | SEA |
| MOL30 | Glycycoumarin | Tyrosine-protein phosphatase non-receptor type 1 | PTPN1 | SEA |
| MOL31 | Glycyrol | ATP-binding cassette sub-family G member 2 | ABCG2 | SEA |
| MOL31 | Glycyrol | Cyclin-A2 | CCNA2 | TCMSP |
| MOL31 | Glycyrol | Cyclic AMP-responsive element-binding protein 1 | CREB1 | SEA |
| MOL31 | Glycyrol | Cytochrome P450 1B1 | CYP1B1 | SEA |
| MOL31 | Glycyrol | Estrogen receptor | ESR1 | TCMSP/SEA |
| MOL31 | Glycyrol | Estrogen receptor beta | ESR2 | SEA |
| MOL31 | Glycyrol | Thrombin | F2 | TCMSP |
| MOL31 | Glycyrol | Glycogen synthase kinase-3 beta | GSK3B | TCMSP |
| MOL31 | Glycyrol | Hypoxia-inducible factor 1-alpha | HIF1A | SEA |
| MOL31 | Glycyrol | Interleukin-2 | IL2 | SEA |
| MOL31 | Glycyrol | Vascular endothelial growth factor receptor 2 | KDR | TCMSP |
| MOL31 | Glycyrol | Mitogen-activated protein kinase 14 | MAPK14 | TCMSP |
| MOL31 | Glycyrol | Nitric oxide synthase, inducible | NOS2 | TCMSP |
| MOL31 | Glycyrol | NAD(P)H dehydrogenase [quinone] 1 | NQO1 | SEA |
| MOL31 | Glycyrol | Proto-oncogene serine/threonine-protein kinase Pim-1 | PIM1 | TCMSP |
| MOL31 | Glycyrol | Peroxisome proliferator activated receptor gamma | PPARG | TCMSP |
| MOL31 | Glycyrol | Prostaglandin G/H synthase 2 | PTGS2 | TCMSP |
| MOL31 | Glycyrol | Tyrosine-protein phosphatase non-receptor type 1 | PTPN1 | SEA |
| MOL31 | Glycyrol | Transcription factor p65 | RELA | SEA |
| MOL32 | Formononetin | Multidrug resistance protein 1 | ABCB1 | SEA |
| MOL32 | Formononetin | ATP-binding cassette sub-family G member 2 | ABCG2 | SEA |
| MOL32 | Formononetin | Acetylcholinesterase | ACHE | TCMSP |
| MOL32 | Formononetin | Androgen receptor | AR | TCMSP |
| MOL32 | Formononetin | Cyclin-A2 | CCNA2 | TCMSP |
| MOL32 | Formononetin | Cell division protein kinase 2 | CDK2 | TCMSP |
| MOL32 | Formononetin | Cytochrome P450 1B1 | CYP1B1 | SEA |
| MOL32 | Formononetin | Dipeptidyl peptidase IV | DPP4 | TCMSP |
| MOL32 | Formononetin | Endothelin receptor type B | EDNRB | SEA |
| MOL32 | Formononetin | Estrogen receptor | ESR1 | TCMSP/SEA |
| MOL32 | Formononetin | Estrogen receptor beta | ESR2 | TCMSP/SEA |
| MOL32 | Formononetin | Steroid hormone receptor ERR1 | ESRRA | SEA |
| MOL32 | Formononetin | Thrombin | F2 | TCMSP |
| MOL32 | Formononetin | Glycogen synthase kinase-3 beta | GSK3B | TCMSP |
| MOL32 | Formononetin | 3 beta-hydroxysteroid dehydrogenase/Delta 5-->4-isomerase type 1 | HSD3B1 | TCMSP |
| MOL32 | Formononetin | Heat shock protein HSP 90 | HSP90AA1 | TCMSP |
| MOL32 | Formononetin | Interleukin-2 | IL2 | SEA |
| MOL32 | Formononetin | Interleukin-4 | IL4 | TCMSP |
| MOL32 | Formononetin | Transcription factor AP-1 | JUN | TCMSP |
| MOL32 | Formononetin | Amine oxidase [flavin-containing] A | MAOA | SEA |
| MOL32 | Formononetin | Mitogen-activated protein kinase 14 | MAPK14 | TCMSP |
| MOL32 | Formononetin | Nitric oxide synthase, inducible | NOS2 | TCMSP |
| MOL32 | Formononetin | Nitric-oxide synthase, endothelial | NOS3 | TCMSP |
| MOL32 | Formononetin | NAD(P)H dehydrogenase [quinone] 1 | NQO1 | SEA |
| MOL32 | Formononetin | [Pyruvate dehydrogenase (acetyl-transferring)] kinase isozyme 4, mitochondrial | PDK4 | SEA |
| MOL32 | Formononetin | Proto-oncogene serine/threonine-protein kinase Pim-1 | PIM1 | TCMSP |
| MOL32 | Formononetin | Peroxisome proliferator activated receptor gamma | PPARG | TCMSP |
| MOL32 | Formononetin | Prostaglandin G/H synthase 1 | PTGS1 | TCMSP |
| MOL32 | Formononetin | Prostaglandin G/H synthase 2 | PTGS2 | TCMSP |
| MOL32 | Formononetin | Retinoic acid receptor RXR-alpha | RXRA | TCMSP |
| MOL32 | Formononetin | NAD-dependent deacetylase sirtuin-1 | SIRT1 | TCMSP |
| MOL33 | Liquiritin apioside | Cytochrome P450 1B1 | CYP1B1 | SEA |
| MOL33 | Liquiritin apioside | Fibroblast growth factor 2 | FGF2 | SEA |
| MOL33 | Liquiritin apioside | Interleukin-2 | IL2 | SEA |
| MOL33 | Liquiritin apioside | Vascular endothelial growth factor A | VEGFA | SEA |
| MOL34 | Isoliquiritin apioside | ATP-binding cassette sub-family G member 2 | ABCG2 | SEA |
| MOL34 | Isoliquiritin apioside | Fibroblast growth factor 2 | FGF2 | SEA |
| MOL34 | Isoliquiritin apioside | Hexokinase-2 | HK2 | SEA |
| MOL34 | Isoliquiritin apioside | Interleukin-2 | IL2 | SEA |
| MOL34 | Isoliquiritin apioside | Sodium/glucose cotransporter 1 | SLC5A1 | SEA |
| MOL34 | Isoliquiritin apioside | Vascular endothelial growth factor A | VEGFA | SEA |
| MOL35 | Licorice-saponin G2 | Aldo-keto reductase family 1 member B10 | AKR1B10 | SEA |
| MOL35 | Licorice-saponin G2 | Tissue factor | F3 | SEA |
| MOL35 | Licorice-saponin G2 | Corticosteroid 11-beta-dehydrogenase isozyme 1 | HSD11B1 | SEA |
| MOL35 | Licorice-saponin G2 | Corticosteroid 11-beta-dehydrogenase isozyme 2 | HSD11B2 | SEA |
| MOL35 | Licorice-saponin G2 | Tyrosine-protein phosphatase non-receptor type 1 | PTPN1 | SEA |
| MOL36 | Glycyrrhizic acid | Aldo-keto reductase family 1 member B10 | AKR1B10 | SEA |
| MOL36 | Glycyrrhizic acid | Tissue factor | F3 | SEA |
| MOL36 | Glycyrrhizic acid | Corticosteroid 11-beta-dehydrogenase isozyme 1 | HSD11B1 | SEA |
| MOL36 | Glycyrrhizic acid | Corticosteroid 11-beta-dehydrogenase isozyme 2 | HSD11B2 | SEA |
| MOL36 | Glycyrrhizic acid | Tyrosine-protein phosphatase non-receptor type 1 | PTPN1 | SEA |
| MOL37 | 3'-Methoxymirificin | Multidrug resistance-associated protein 1 | ABCC1 | SEA |
| MOL37 | 3'-Methoxymirificin | Cytochrome P450 1A1 | CYP1A1 | SEA |
| MOL37 | 3'-Methoxymirificin | Cytochrome P450 1B1 | CYP1B1 | SEA |
| MOL37 | 3'-Methoxymirificin | Interleukin-2 | IL2 | SEA |
| MOL37 | 3'-Methoxymirificin | Interleukin-6 | IL6 | SEA |
| MOL37 | 3'-Methoxymirificin | NADH-ubiquinone oxidoreductase 75 kDa subunit, mitochondrial | NDUFS1 | SEA |
| MOL39 | Ononin | ATP-binding cassette sub-family G member 2 | ABCG2 | SEA |
| MOL39 | Ononin | Aldehyde dehydrogenase, mitochondrial | ALDH2 | SEA |
| MOL39 | Ononin | Androgen receptor | AR | TCMSP |
| MOL39 | Ononin | Cytochrome P450 1B1 | CYP1B1 | SEA |
| MOL39 | Ononin | Dipeptidyl peptidase IV | DPP4 | TCMSP |
| MOL39 | Ononin | Estrogen receptor | ESR1 | TCMSP |
| MOL39 | Ononin | Thrombin | F2 | TCMSP |
| MOL39 | Ononin | Fibroblast growth factor 2 | FGF2 | SEA |
| MOL39 | Ononin | Glyceraldehyde-3-phosphate dehydrogenase | GAPDH | SEA |
| MOL39 | Ononin | Interleukin-2 | IL2 | SEA |
| MOL39 | Ononin | Interleukin-6 | IL6 | SEA |
| MOL39 | Ononin | Vascular endothelial growth factor receptor 2 | KDR | TCMSP |
| MOL39 | Ononin | Nitric oxide synthase, inducible | NOS2 | TCMSP |
| MOL39 | Ononin | Peroxisome proliferator activated receptor gamma | PPARG | TCMSP |
| MOL39 | Ononin | Prostaglandin G/H synthase 2 | PTGS2 | TCMSP |
| MOL39 | Ononin | Sodium/glucose cotransporter 1 | SLC5A1 | SEA |
| MOL39 | Ononin | Sodium/glucose cotransporter 2 | SLC5A2 | SEA |
| MOL39 | Ononin | Solute carrier family 5 member 4 | SLC5A4 | SEA |
| MOL39 | Ononin | Vascular endothelial growth factor A | VEGFA | SEA |
| MOL40 | Glycyrrhetinic acid | Aldo-keto reductase family 1 member B10 | AKR1B10 | SEA |
| MOL40 | Glycyrrhetinic acid | Catenin beta-1 | CTNNB1 | TCMSP |
| MOL40 | Glycyrrhetinic acid | Cytochrome P450 2E1 | CYP2E1 | TCMSP |
| MOL40 | Glycyrrhetinic acid | Tissue factor | F3 | SEA |
| MOL40 | Glycyrrhetinic acid | Gap junction alpha-1 protein | GJA1 | TCMSP |
| MOL40 | Glycyrrhetinic acid | G-protein coupled bile acid receptor 1 | GPBAR1 | SEA |
| MOL40 | Glycyrrhetinic acid | Corticosteroid 11-beta-dehydrogenase isozyme 1 | HSD11B1 | SEA |
| MOL40 | Glycyrrhetinic acid | Corticosteroid 11-beta-dehydrogenase isozyme 2 | HSD11B2 | TCMSP/SEA |
| MOL40 | Glycyrrhetinic acid | Interleukin-1 beta | IL1B | SEA |
| MOL40 | Glycyrrhetinic acid | Tyrosine-protein phosphatase non-receptor type 1 | PTPN1 | SEA |
| MOL42 | Chrysin 7-O-glucuronide | Multidrug resistance protein 1 | ABCB1 | SEA |
| MOL42 | Chrysin 7-O-glucuronide | Multidrug resistance-associated protein 1 | ABCC1 | SEA |
| MOL42 | Chrysin 7-O-glucuronide | ATP-binding cassette sub-family G member 2 | ABCG2 | SEA |
| MOL42 | Chrysin 7-O-glucuronide | Acetylcholinesterase | ACHE | TCMSP |
| MOL42 | Chrysin 7-O-glucuronide | Aldose reductase | AKR1B1 | SEA |
| MOL42 | Chrysin 7-O-glucuronide | Aldo-keto reductase family 1 member B10 | AKR1B10 | SEA |
| MOL42 | Chrysin 7-O-glucuronide | Aldehyde dehydrogenase, mitochondrial | ALDH2 | SEA |
| MOL42 | Chrysin 7-O-glucuronide | Cyclic AMP-responsive element-binding protein 1 | CREB1 | SEA |
| MOL42 | Chrysin 7-O-glucuronide | Cytochrome P450 1A1 | CYP1A1 | SEA |
| MOL42 | Chrysin 7-O-glucuronide | Cytochrome P450 1B1 | CYP1B1 | SEA |
| MOL42 | Chrysin 7-O-glucuronide | Dipeptidyl peptidase IV | DPP4 | TCMSP |
| MOL42 | Chrysin 7-O-glucuronide | Thrombin | F2 | TCMSP |
| MOL42 | Chrysin 7-O-glucuronide | Fibroblast growth factor 2 | FGF2 | SEA |
| MOL42 | Chrysin 7-O-glucuronide | Interleukin-2 | IL2 | SEA |
| MOL42 | Chrysin 7-O-glucuronide | Interleukin-6 | IL6 | SEA |
| MOL42 | Chrysin 7-O-glucuronide | Amine oxidase [flavin-containing] A | MAOA | TCMSP |
| MOL42 | Chrysin 7-O-glucuronide | Nitric-oxide synthase, brain | NOS1 | TCMSP |
| MOL42 | Chrysin 7-O-glucuronide | Nitric oxide synthase, inducible | NOS2 | TCMSP |
| MOL42 | Chrysin 7-O-glucuronide | Nitric-oxide synthase, endothelial | NOS3 | TCMSP |
| MOL42 | Chrysin 7-O-glucuronide | NADPH oxidase 4 | NOX4 | SEA |
| MOL42 | Chrysin 7-O-glucuronide | Placenta growth factor | PGF | SEA |
| MOL42 | Chrysin 7-O-glucuronide | Prostaglandin G/H synthase 1 | PTGS1 | TCMSP |
| MOL42 | Chrysin 7-O-glucuronide | Prostaglandin G/H synthase 2 | PTGS2 | TCMSP |
| MOL42 | Chrysin 7-O-glucuronide | Sodium/glucose cotransporter 1 | SLC5A1 | SEA |
| MOL42 | Chrysin 7-O-glucuronide | Sodium/glucose cotransporter 2 | SLC5A2 | SEA |
| MOL42 | Chrysin 7-O-glucuronide | Solute carrier family 5 member 4 | SLC5A4 | SEA |
| MOL42 | Chrysin 7-O-glucuronide | Transthyretin | TTR | SEA |
| MOL42 | Chrysin 7-O-glucuronide | Vascular endothelial growth factor A | VEGFA | SEA |
| MOL42 | Chrysin 7-O-glucuronide | Xanthine dehydrogenase/oxidase | XDH | SEA |

Supplementary Table S3 The SMILES structural similarity

| Number1 | Compounds | SMILES |
| --- | --- | --- |
| MOL01 | Daidzin | C1=CC(=CC=C1C2=COC3=C(C2=O)C=CC(=C3)OC4C(C(C(C(O4)CO)O)O)O)O |
| MOL02 | 3-Methoxypuerarin | COC1=C(C=CC(=C1)C2=COC3=C(C2=O)C=CC(=C3C4C(C(C(C(O4)CO)O)O)O)O)O |
| MOL03 | Daidzein | C1=CC(=CC=C1C2=COC3=C(C2=O)C=CC(=C3)O)O |
| MOL04 | Genistin | C1=CC(=CC=C1C2=COC3=CC(=CC(=C3C2=O)O)OC4C(C(C(C(O4)CO)O)O)O)O |
| MOL05 | Formononetin 8-C-apiofuranosyl (1,6)glucoside | NA |
| MOL06 | Genistein 8-C-apiofuranosyl(1,6)glucoside | NA |
| MOL07 | Puerarin | C1=CC(=CC=C1C2=COC3=C(C2=O)C=CC(=C3C4C(C(C(C(O4)CO)O)O)O)O)O |
| MOL08 | Wogonin | COC1=C(C=C(C2=C1OC(=CC2=O)C3=CC=CC=C3)O)O |
| MOL09 | Oroxylin A | COC1=C(C2=C(C=C1O)OC(=CC2=O)C3=CC=CC=C3)O |
| MOL10 | Chrysin 6-C-arabinoside-8-C-glucoside | C1C(C(C(C(O1)C2=C(C(=C3C(=C2O)C(=O)C=C(O3)C4=CC=CC=C4)C5C(C(C(C(O5)CO)O)O)O)O)O)O)O |
| MOL11 | Chrysin-8-C-arabinoside-6-C-glucoside | C1C(C(C(C(O1)C2=C3C(=C(C(=C2O)C4C(C(C(C(O4)CO)O)O)O)O)C(=O)C=C(O3)C5=CC=CC=C5)O)O)O |
| MOL12 | Baicalein | C1=CC=C(C=C1)C2=CC(=O)C3=C(O2)C=C(C(=C3O)O)O |
| MOL13 | Wogonin 5-O-glucoside | COC1=C2C(=C(C=C1O)O[C@H]3C([C@H]([C@@H](C(O3)CO)O)O)O)C(=O)C=C(O2)C4=CC=CC=C4 |
| MOL14 | Norwogonin 7-O-glucuronide | C1=CC=C(C=C1)C2=CC(=O)C3=C(O2)C(=C(C=C3O)OC4C(C(C(C(O4)C(=O)O)O)O)O)O |
| MOL15 | Oroxylin A 7-O-glucuronide | COC1=C(C=C2C(=C1O)C(=O)C=C(O2)C3=CC=CC=C3)OC4C(C(C(C(O4)C(=O)O)O)O)O |
| MOL16 | Wogonoside | COC1=C(C=C(C2=C1OC(=CC2=O)C3=CC=CC=C3)O)O[C@H]4[C@@H]([C@H]([C@@H]([C@H](O4)C(=O)O)O)O)O |
| MOL17 | Baicalin | C1=CC=C(C=C1)C2=CC(=O)C3=C(C(=C(C=C3O2)OC4C(C(C(C(O4)C(=O)O)O)O)O)O)O |
| MOL18 | Chrysin | C1=CC=C(C=C1)C2=CC(=O)C3=C(C=C(C=C3O2)O)O |
| MOL19 | Magnoflorine | C[N+]1(CCC2=CC(=C(C3=C2C1CC4=C3C(=C(C=C4)OC)O)O)OC)C |
| MOL20 | Demethyleneberberine | COC1=C(C2=C[N+]3=C(C=C2C=C1)C4=CC(=C(C=C4CC3)O)O)OC |
| MOL21 | Coptisine | C1C[N+]2=C(C=C3C=CC4=C(C3=C2)OCO4)C5=CC6=C(C=C51)OCO6 |
| MOL22 | Epiberberine | COC1=C(C=C2C(=C1)CC[N+]3=C2C=C4C=CC5=C(C4=C3)OCO5)OC |
| MOL23 | Jatrorrhizine | COC1=C(C2=C[N+]3=C(C=C2C=C1)C4=CC(=C(C=C4CC3)O)OC)OC |
| MOL24 | Berberine | COC1=C(C2=C[N+]3=C(C=C2C=C1)C4=CC5=C(C=C4CC3)OCO5)OC |
| MOL25 | Palmatine | COC1=C(C2=C[N+]3=C(C=C2C=C1)C4=CC(=C(C=C4CC3)OC)OC)OC |
| MOL26 | Liquiritin | c1cc(ccc1[C@@H]2CC(=O)c3ccc(cc3O2)O)O[C@H]4[C@@H]([C@H]([C@@H]([C@H](O4)CO)O)O)O |
| MOL27 | Isoliquiritin | C1=CC(=CC=C1C=CC(=O)C2=C(C=C(C=C2)O)O)OC3C(C(C(C(O3)CO)O)O)O |
| MOL28 | Isoliquiritigenin | C1=CC(=CC=C1/C=C/C(=O)C2=C(C=C(C=C2)O)O)O |
| MOL29 | Liquiritigenin | C1C(OC2=C(C1=O)C=CC(=C2)O)C3=CC=C(C=C3)O |
| MOL30 | Glycycoumarin | CC(=CCC1=C(C2=C(C=C1O)OC(=O)C(=C2)C3=C(C=C(C=C3)O)O)OC)C |
| MOL31 | Glycyrol | CC(=CCC1=C(C2=C(C=C1O)OC(=O)C3=C2OC4=C3C=CC(=C4)O)OC)C |
| MOL32 | Formononetin | COC1=CC=C(C=C1)C2COC3=C(C2=O)C=CC(=C3)O |
| MOL33 | Liquiritin apioside | c1cc(ccc1[C@@H]2CC(=O)c3ccc(cc3O2)O)O[C@H]4[C@@H]([C@H]([C@@H]([C@H](O4)CO)O)O)O[C@H]5[C@@H]([C@](CO5)(CO)O)O |
| MOL34 | Isoliquiritin apioside | c1cc(ccc1/C=C/C(=O)c2ccc(cc2O)O)O[C@H]3[C@@H]([C@H]([C@@H]([C@H](O3)CO)O)O)O[C@H]4[C@@H]([C@](CO4)(CO)O)O |
| MOL35 | Licorice-saponin G2 | CC12CCC(CC1C3=CC(=O)C4C5(CCC(C(C5CCC4(C3(CC2)C)C)(C)CO)OC6C(C(C(C(O6)C(=O)O)O)O)OC7C(C(C(C(O7)C(=O)O)O)O)O)C)(C)C(=O)O |
| MOL36 | Glycyrrhizic acid | CC1(C2CCC3(C(C2(CCC1OC4C(C(C(C(O4)C(=O)O)O)O)OC5C(C(C(C(O5)C(=O)O)O)O)O)C)C(=O)C=C6C3(CCC7(C6CC(CC7)(C)C(=O)O)C)C)C)C |
| MOL37 | 3'-Methoxymirificin | COC1=C(C=CC(=C1)C2=CC(=O)C3=C(O2)C(=C(C=C3)O)C4C(C(C(C(O4)COC5C(C(CO5)(CO)O)O)O)O)O)O |
| MOL38 | Lateriflorein 7-O-glucuronide | NA |
| MOL39 | Ononin | COC1=CC=C(C=C1)C2=COC3=C(C2=O)C=CC(=C3)OC4C(C(C(C(O4)CO)O)O)O |
| MOL40 | Glycyrrhetinic acid | CC1(C2CCC3(C(C2(CCC1O)C)C(=O)C=C4C3(CCC5(C4CC(CC5)(C)C(=O)O)C)C)C)C |
| MOL41 | (4S)-Puerol B 2''-O-glucopyranoside | NA |
| MOL42 | Chrysin 7-O-glucuronide | C1=CC=C(C=C1)C2=CC(=O)C3=C(C=C(C=C3O2)OC4C(C(C(C(O4)CO)O)O)O)O |

Supplementary Table S4 T2DM-related target genes

| **Number** | **Protein name** | **Gene name** | **Degree** |
| --- | --- | --- | --- |
| 1 | Cytochrome P450 1B1 | CYP1B1 | 30 |
| 2 | Interleukin-2 | IL2 | 23 |
| 3 | ATP-binding cassette sub-family G member 2 | ABCG2 | 21 |
| 4 | Prostaglandin G/H synthase 2 | PTGS2 | 20 |
| 5 | Vascular endothelial growth factor A | VEGFA | 19 |
| 6 | Estrogen receptor | ESR1 | 17 |
| 7 | Xanthine dehydrogenase/oxidase | XDH | 17 |
| 8 | Androgen receptor | AR | 16 |
| 9 | Multidrug resistance protein 1 | ABCB1 | 14 |
| 10 | Aldo-keto reductase family 1 member B10 | AKR1B10 | 14 |
| 11 | Nitric oxide synthase, inducible | NOS2 | 14 |
| 12 | Prostaglandin G/H synthase 1 | PTGS1 | 14 |
| 13 | Aldose reductase | AKR1B1 | 14 |
| 14 | Cyclic AMP-responsive element-binding protein 1 | CREB1 | 14 |
| 15 | Cytochrome P450 1A1 | CYP1A1 | 14 |
| 16 | Interleukin-6 | IL6 | 13 |
| 17 | Solute carrier family 5 member 4 | SLC5A4 | 12 |
| 18 | Heat shock protein HSP 90 | HSP90AA1 | 12 |
| 19 | Fibroblast growth factor 2 | FGF2 | 11 |
| 20 | Sodium/glucose cotransporter 1 | SLC5A1 | 11 |
| 21 | Estrogen receptor beta | ESR2 | 11 |
| 22 | Cyclin-A2 | CCNA2 | 11 |
| 23 | Sodium/glucose cotransporter 2 | SLC5A2 | 10 |
| 24 | mRNA of Protein-tyrosine phosphatase, non-receptor type 1 | PTPN1 | 10 |
| 25 | Nitric oxide synthase, endothelial | NOS3 | 10 |
| 26 | Placenta growth factor | PGF | 10 |
| 27 | Retinoic acid receptor RXR-alpha | RXRA | 10 |
| 28 | Amine oxidase [flavin-containing] A | MAOA | 9 |
| 29 | NADPH oxidase 4 | NOX4 | 9 |
| 30 | Multidrug resistance-associated protein 1 | ABCC1 | 9 |
| 31 | Dipeptidyl peptidase IV | DPP4 | 8 |
| 32 | Thrombin | F2 | 8 |
| 33 | Peroxisome proliferator activated receptor gamma | PPARG | 8 |
| 34 | NAD(P)H dehydrogenase [quinone] 1 | NQO1 | 8 |
| 35 | Acetylcholinesterase | ACHE | 7 |
| 36 | Aldehyde dehydrogenase, mitochondrial | ALDH2 | 7 |
| 37 | Glycogen synthase kinase-3 beta | GSK3B | 7 |
| 38 | Proto-oncogene serine/threonine-protein kinase Pim-1 | PIM1 | 7 |
| 39 | Cell division protein kinase 2 | CDK2 | 6 |
| 40 | Transthyretin | TTR | 6 |
| 41 | Phosphatidylinositol-4,5-bisphosphate 3-kinase catalytic subunit, gamma isoform | PIK3CG | 6 |
| 42 | Tissue factor | F3 | 6 |
| 43 | Ras-related C3 botulinum toxin substrate 1 | RAC1 | 6 |
| 44 | Aryl hydrocarbon receptor | AHR | 5 |
| 45 | Apoptosis regulator BAX | BAX | 5 |
| 46 | Caspase-3 | CASP3 | 5 |
| 47 | Steroid hormone receptor ERR1 | ESRRA | 5 |
| 48 | Transcription factor AP-1 | JUN | 5 |
| 49 | Mitogen-activated protein kinase 14 | MAPK14 | 5 |
| 50 | Transcription factor p65 | RELA | 5 |
| 51 | Hypoxia-inducible factor 1-alpha | HIF1A | 5 |
| 52 | Cytochrome P450 1A2 | CYP1A2 | 5 |
| 53 | Coagulation factor Xa | F10 | 4 |
| 54 | Vascular endothelial growth factor receptor 2 | KDR | 4 |
| 55 | Cyclin-dependent kinase inhibitor 1 | CDKN1B | 4 |
| 56 | Proto-oncogene c-Fos | FOS | 4 |
| 57 | Myeloperoxidase | MPO | 4 |
| 58 | Apoptosis regulator Bcl-2 | BCL2 | 4 |
| 59 | G2/mitotic-specific cyclin-B1 | CCNB1 | 4 |
| 60 | Nuclear receptor subfamily 4 group A member 2 | NR4A2 | 4 |
| 61 | Amyloid-beta A4 protein | APP | 4 |
| 62 | Hexokinase-2 | HK2 | 4 |
| 63 | Interleukin-4 | IL4 | 3 |
| 64 | Serum paraoxonase/arylesterase 1 | PON1 | 3 |
| 65 | Tumor necrosis factor | TNF | 3 |
| 66 | Cellular tumor antigen p53 | TP53 | 3 |
| 67 | Vascular cell adhesion protein 1 | VCAM1 | 3 |
| 68 | RAC-alpha serine/threonine-protein kinase | AKT1 | 3 |
| 69 | Matrix metalloproteinase-9 | MMP9 | 3 |
| 70 | Cyclin-dependent kinase 1 | CDK1 | 3 |
| 71 | D(1A) dopamine receptor | DRD1 | 3 |
| 72 | [Pyruvate dehydrogenase (acetyl-transferring)] kinase isozyme 4, mitochondrial | PDK4 | 3 |
| 73 | Corticosteroid 11-beta-dehydrogenase isozyme 1 | HSD11B1 | 3 |
| 74 | Corticosteroid 11-beta-dehydrogenase isozyme 2 | HSD11B2 | 3 |
| 75 | Cytochrome P450 2C9 | CYP2C9 | 2 |
| 76 | Cytochrome P450 3A4 | CYP3A4 | 2 |
| 77 | 3 beta-hydroxysteroid dehydrogenase/Delta 5-->4-isomerase type 1 | HSD3B1 | 2 |
| 78 | Metallothionein-2 | MT2A | 2 |
| 79 | Bcl2 antagonist of cell death | BAD | 2 |
| 80 | Caspase-9 | CASP9 | 2 |
| 81 | 72 kDa type IV collagenase | MMP2 | 2 |
| 82 | Interstitial collagenase | MMP1 | 2 |
| 83 | Glutathione S-transferase A1 | GSTA1 | 2 |
| 84 | Beta-secretase 1 | BACE1 | 2 |
| 85 | Microtubule-associated protein tau | MAPT | 2 |
| 86 | Apolipoprotein B-100 | APOB | 1 |
| 87 | Catalase | CAT | 1 |
| 88 | Caveolin-1 | CAV1 | 1 |
| 89 | Carnitine O-palmitoyltransferase 1, liver isoform | CPT1A | 1 |
| 90 | Histone acetyltransferase p300 | EP300 | 1 |
| 91 | Growth arrest and DNA damage-inducible protein GADD45 alpha | GADD45A | 1 |
| 92 | Intercellular adhesion molecule 1 | ICAM1 | 1 |
| 93 | Insulin-like growth factor IA | IGF1 | 1 |
| 94 | Insulin-like growth factor 1 receptor | IGF1R | 1 |
| 95 | Low-density lipoprotein receptor | LDLR | 1 |
| 96 | Antigen KI-67 | MKI67 | 1 |
| 97 | Microsomal triglyceride transfer protein large subunit | MTTP | 1 |
| 98 | Superoxide dismutase [Mn], mitochondrial | SOD2 | 1 |
| 99 | Angiotensin-converting enzyme 2 | ACE2 | 1 |
| 100 | Baculoviral IAP repeat-containing protein 5 | BIRC5 | 1 |
| 101 | Caspase-8 | CASP8 | 1 |
| 102 | Alanine aminotransferase 1 | GPT | 1 |
| 103 | Glutathione S-transferase P | GSTP1 | 1 |
| 104 | Interferon beta | IFNB1 | 1 |
| 105 | Leptin receptor | LEPR | 1 |
| 106 | Mitogen-activated protein kinase 9 | MAPK9 | 1 |
| 107 | NF-kappa-B inhibitor alpha | NFKBIA | 1 |
| 108 | Tissue-type plasminogen activator | PLAT | 1 |
| 109 | Perilipin-2 | PLIN2 | 1 |
| 110 | Protein kinase C alpha type | PRKCA | 1 |
| 111 | Superoxide dismutase [Cu-Zn] | SOD1 | 1 |
| 112 | Signal transducer and activator of transcription 3 | STAT3 | 1 |
| 113 | Metalloproteinase inhibitor 2 | TIMP2 | 1 |
| 114 | C-C motif chemokine 2 | CCL2 | 1 |
| 115 | G1/S-specific cyclin-D1 | CCND1 | 1 |
| 116 | G1/S-specific cyclin-E1 | CCNE1 | 1 |
| 117 | Interleukin-8 | CXCL8 | 1 |
| 118 | Fibronectin | FN1 | 1 |
| 119 | Beta-glucuronidase | GUSB | 1 |
| 120 | Protein kinase C delta type | PRKCD | 1 |
| 121 | Cytochrome c | CYCS | 1 |
| 122 | Fos-related antigen 1 | FOSL1 | 1 |
| 123 | Insulin-like growth factor II | IGF2 | 1 |
| 124 | Mothers against decapentaplegic homolog 3 | SMAD3 | 1 |
| 125 | Epidermal growth factor receptor | EGFR | 1 |
| 126 | Interleukin-13 | IL13 | 1 |
| 127 | Transforming growth factor beta-1 | TGFB1 | 1 |
| 128 | Pregnane X receptor | NR1I2 | 1 |
| 129 | Histone deacetylase 1 | HDAC1 | 1 |
| 130 | 60 kDa heat shock protein, mitochondrial | HSPD1 | 1 |
| 131 | Tyrosine-protein kinase JAK2 | JAK2 | 1 |
| 132 | Nuclear factor erythroid 2-related factor 2 | NFE2L2 | 1 |
| 133 | Nuclear factor NF-kappa-B p105 subunit | NFKB1 | 1 |
| 134 | Nuclear receptor subfamily 0 group B member 2 | NR0B2 | 1 |
| 135 | [Pyruvate dehydrogenase (acetyl-transferring)] kinase isozyme 1, mitochondrial | PDK1 | 1 |
| 136 | E-selectin | SELE | 1 |
| 137 | Solute carrier family 2, facilitated glucose transporter member 1 | SLC2A1 | 1 |
| 138 | Tumor necrosis factor receptor superfamily member 1A | TNFRSF1A | 1 |
| 139 | Endothelin receptor type B | EDNRB | 1 |
| 140 | NAD-dependent deacetylase sirtuin-1 | SIRT1 | 1 |
| 141 | NADH-ubiquinone oxidoreductase 75 kDa subunit, mitochondrial | NDUFS1 | 1 |
| 142 | Glyceraldehyde-3-phosphate dehydrogenase | GAPDH | 1 |
| 143 | Catenin beta-1 | CTNNB1 | 1 |
| 144 | Cytochrome P450 2E1 | CYP2E1 | 1 |
| 145 | Gap junction alpha-1 protein | GJA1 | 1 |
| 146 | G-protein coupled bile acid receptor 1 | GPBAR1 | 1 |
| 147 | Interleukin-1 beta | IL1B | 1 |
| 148 | Nitric-oxide synthase, brain | NOS1 | 1 |
